# Microarray and Morphological Analysis of Early Postnatal CRB2 Mutant Retinas on a Pure C57BL/6J Genetic Background

**DOI:** 10.1371/journal.pone.0082532

**Published:** 2013-12-06

**Authors:** Celso Henrique Alves, Koen Bossers, Rogier M. Vos, Anke H. W. Essing, Sigrid Swagemakers, Peter J. van der Spek, Joost Verhaagen, Jan Wijnholds

**Affiliations:** 1 Department of Neuromedical Genetics, The Netherlands Institute for Neuroscience, Royal Netherlands Academy of Arts and Sciences, Amsterdam, The Netherlands; 2 Department of Neuroregeneration, The Netherlands Institute for Neuroscience, Royal Netherlands Academy of Arts and Sciences, Amsterdam, The Netherlands; 3 Department of Bioinformatics, Erasmus University Medical Centre, Rotterdam, The Netherlands; University of Cologne, Germany

## Abstract

In humans, the Crumbs homologue-1 (*CRB1*) gene is mutated in progressive types of autosomal recessive retinitis pigmentosa and Leber congenital amaurosis. The severity of the phenotype due to human *CRB1* or mouse *Crb1* mutations is dependent on the genetic background. Mice on C57BL/6J background with *Crb1* mutations show late onset of retinal spotting phenotype or no phenotype. Recently, we showed that conditional deletion of mouse *Crb2* in the retina results in early retinal disorganization leading to severe and progressive retinal degeneration with concomitant visual loss that mimics retinitis pigmentosa due to mutations in the *CRB1* gene. Recent studies in the fruit fly and zebrafish suggest roles of the Crumbs (CRB) complex members in the regulation of cellular signalling pathways including the Notch1, mechanistic target of rapamycin complex 1 (mTORC1) and the Hippo pathway. Here, we demonstrate that mice backcrossed to C57BL/6J background with loss of CRB2 in the retina show a progressive disorganization and degeneration phenotype during late retinal development. We used microarray gene profiling to study the transcriptome of retinas lacking CRB2 during late retinal development. Unexpectedly, the retinas of newborn mice lacking CRB2 showed no changes in the transcriptome during retinal development. These findings suggest that loss of CRB2 in the developing retina results in retinal disorganization and subsequent degeneration without major changes in the transcriptome of the retina. These mice might be an interesting model to study the onset of retinal degeneration upon loss of CRB proteins.

## Introduction

Retinal cell generation and differentiation in the mouse occurs from embryonic day (E) 11 to postnatal day (P) 10. Six major neuronal and one glia cell type are generated from multipotential retinal progenitors in a characteristic sequence during development [1,2]. Cell adhesion and cell polarity protein complexes, such as the Crumbs (CRB) and adherens junctions complexes, play a critical role in maintenance of the proliferation of the progenitor cells [3]. Changes in these complexes disturb the spatiotemporal aspects of retinogenesis, leading to retinal degeneration resulting in mild or severe impairment of retinal function and vision [4-8]. Recent studies demonstrated that the CRB complex members are able to regulate several important signalling pathways including the Notch1 [9-11], mechanistic target of rapamycin complex 1 (mTORC1) [12,13] and the Hippo pathway [3,14-17].

 The apical CRB complex is located at the subapical region adjacent to adherens junctions between the retinal progenitor cells in the developing retina [4] and, after differentiation, at the subapical regions of Müller glia and photoreceptor cells [7,18]. In mammals, the CRB family consists of CRB1, CRB2, CRB3A and CRB3B [19].

In humans, mutations in the *CRB1* gene are responsible for retinal diseases such as Leber congenital amaurosis and retinitis pigmentosa [19-21]. The lack of a clear genotype–phenotype correlation suggests that other components of the CRB complex have a function influencing the severity of the retinal disease. We recently showed that conditional deletion of the *Crb* gene family member *Crb2*, specifically in early progenitors, results in disorganization during late retinal development leading to severe and progressive retinal degeneration with concomitant visual loss that mimics retinitis pigmentosa due to mutations in the *CRB1* gene [4]. Variation in the genetic background may influence the severity of the retinal phenotype, as described before for the *Crb1* knockout (*Crb1* ^*-/-*^) and *Crb1Crb2* conditional knockout mice [7,22,23].

Microarray analysis has been used to study changes in gene expression during retinal development [24-29]. Many of the previous studies using this technique focused on a specific cell type, for example using isolated single cells [30] or cell populations [31], or retinas from cell type specific mutants [32-36]. Backcrossing *Crb1* mutant retinas from mixed to C57BL/6J genetic background strongly suppressed the morphological phenotype [22]. Here, we show that backcrossing *Crb2* null retinas from mixed (50% OLA129 and 50% C57BL/6J) to 99.9% C57BL/6J background did not suppress the severe morphological phenotype. To elucidate the molecular events that precede and lead to the morphological phenotype in the *Crb2* retina-specific conditional knockout mice on 99.9% C57BL/6J background, we applied microarray-based mRNA profiling in retinal tissue of postnatal stages P0, P3, P6, and P10. The morphological phenotype did however not result in a significantly altered transcriptome at any stage of retinal development analysed.

## Materials and Methods

### Animals

All procedures concerning animals were performed with permission of the animal experimentation committee (DEC) of the Royal Netherlands Academy of Arts and Sciences (KNAW), permit number NIN06-46. *Crb2* conditional knockout mice were generated and genotyped as described in [4], and were backcrossed to 99.9% C57BL/6J genetic background. Mice had no mutations in the phosphodiesterase 6b (*pde6b*) or *Crb1* (*rd8*) genes. mT/mG reporter mice were obtained from the Jackson laboratory (Gt(ROSA)26Sortm4(ACTB-tdTomato,-EGFP)) [37]. Animals were maintained on a 12 h day/night cycle and supplied with food and water *ad libitum*. 

### Tissue collection for RNA isolation

Pups were euthanized by decapitation at different time points: postnatal day (P) 0, P3, P6 and P10. Five control (*Crb2F* ^/+^/Chx10Cre^+/-^) and five *Crb2*Chx10 cKO (*Crb2F/F/*Chx10*Cre* ^*+/-*^) retinas per time point were used. The retinas were isolated, snap-frozen in liquid nitrogen immediately, and stored at -80°C until use. 

### RNA isolation and amplification

Retinae were thawed in RLT buffer (RNeasy Mini Kit, catalogue number: 74106; Qiagen Benelux, Venlo, The Netherlands) and homogenized with a pellet pestle. Total RNA was isolated using RNeasy Mini Kit according to the manufacturer's instructions. RNA extract was eluted in RNase-free water (30 μL). RNA yield and purity were determined using a NanoDrop ND-1000 spectrophotometer (NanoDrop Technologies, Wilmington, DE, USA). RNA integrity was determined by the RNA integrity number (RIN), as measured by the Agilent 2100 bioanalyser (Agilent Technologies, Palo Alto, CA, USA). Overall, the isolated RNA was of high integrity (average RIN = 9.3, range 8.3–10.0, Table S1 and Table S2).

### Sample labelling and microarray hybridization

For microarray analysis, Agilent 4x44K v2 whole mouse gene expression microarrays (Agilent Technologies, catalogue number G4846A) were used. Sample labelling and microarray hybridization were performed according to manufacturer’s instructions. Briefly, for each sample, 200 ng of RNA were linearly amplified and fluorescently labelled with either Cy3-CTP or Cy5-CTP (Perkin Elmer) using the Agilent Low RNA Input Fluorescent Linear Amplification Kit (Agilent Technologies). Prior to hybridization, equal amounts (825 ng) of Cy3- and Cy5-labelled RNA were hydrolyzed for 30 min at 60°C in 1x fragmentation buffer (Agilent Technologies). The fragmented targets were hybridized to a microarray by incubating for 17 h at 60°C in 1x target solution (Agilent Technologies) in a rotating hybridization chamber. Specifically, each hybridization consisted of two individual samples (one control and one knockout), one labelled with Cy3 and one with Cy5. None of the samples were pooled. A full description of all hybridizations can be found in Table S3. After hybridization, the arrays were washed at room temperature for 5 min in 6x saline-sodium phosphate-EDTA (SSPE)/0.005% *N*-Lauroylsarcosine (Sigma-Aldrich, St Louis, MO, USA), 1 min in 0.06x SSPE/0.005% *N*-Lauroylsarcosine and 30 s in acetonitrile (Sigma-Aldrich), then dried in a nitrogen flow. Microarrays were scanned using an Agilent DNA Microarray Scanner at 5 µm resolution and 10% (low) and 100% (high) photomultiplier tube setting. Microarray scans were quantified using Agilent Feature Extraction software (version 9.5.3.1).

### Microarray normalization and single gene analysis

Raw expression data were analyzed in R statistical processing software (version 2.6.0) using the LIMMA package [38] in Bioconductor (www.bioconductor.org). All features that were flagged as saturated or as a nonuniformity outlier by the feature extraction software on 1 or more arrays were excluded from further analysis. This was applicable for 980 features, resulting in 43,020 features which passed these criteria. Array 20 was excluded from the studies due to a technical artefact. Data were normalized using LIMMA by applying a background correction (using the ‘normexp’ algorithm) followed by normalization of intensity distributions within and between arrays (using the ‘quantile’ algorithm). It was recently demonstrated that the analysis of the separate intensity channels (the individual Cy3 and Cy5 signals) yields more reproducible results than the standard ratio-based approach (the ratio between the Cy3 and Cy5 channels) for dual-color microarray datasets [39], so we also applied this approach for the present dataset. Thus, the ^2^log-transformed intensity measurements per sample were extracted from the normalized ratio data and used in all following analyses.

To detect genes with a significant interaction between age and genotype, we performed a 2-way analysis of variance (ANOVA) using age and genotype as grouping factors. To compare gene expression between *Crb2* cKO and control retinas at each timepoint separately t-tests were used. The Benjamini–Hochberg method was used to correct for multiple testing. *P*-values < 0.05 after correction were considered significant. 

A second and independent analysis of the data was done using Partek version 6.6 (Partek Inc., St. Louis, MO). The Feature Extraction result text files were imported into Partek, log_2_ transformed and quantile normalized. To visualize the clustering of the samples, principal component analysis (PCA) was used. Differentially expressed genes were selected using a 2-way ANOVA using the factors genotype and age. The cutoff value for significantly expressed genes was a false discovery rate (FDR) [40], of 0.05 or less.

The data discussed in this publication have been deposited in NCBI's Gene Expression Omnibus [41] and are accessible through GEO Series accession number GSE50845 (http://www.ncbi.nlm.nih.gov/geo/query/acc.cgi?acc=GSE50845).

### Quantitative real-time PCR

RNA was isolated, from P3 and P10 retinas, 5-6 control and *Crb2* cKO, as indicated before. After genomic DNA degradation with RNase-free DNase I (New England Biolabs), 0.5 µg of total RNA was reverse transcribed into first-strand cDNA with Superscript III Plus RNase H-Reverse Transcriptase (Life Technologies) and 50 ng random hexamer primers, during 50 min at 50°C in a total volume of 20 µl. To the resulting cDNA sample, 14 µl of 10 mM Tris, 1 mM EDTA was added. From all samples, a 1:20 dilution was made and used for qPCR analysis. For this analysis, primer pairs were designed with a melting temperature of 60 °C, giving rise to an amplicon of 60–206 bp. Real-time qPCR was based on the real-time monitoring of SYBR Green I dye fluorescence on a ABI Prism 7300 Sequence Detection System (Applied Biosystems, Nieuwekerk a/d IJssel, The Netherlands). The PCR conditions were as follows: 12.5 µL SYBR Green PCR 2x mastermix (Applied Biosystems), 20 pmol of primers (see Table S4), and 2 µl of the diluted cDNA (ca 3 ng total RNA input). An initial step of 50°C for 2 min was used for AmpErase incubation followed by 15 min at 95°C to inactivate AmpErase and to activate the AmpliTaq. Cycling conditions were as follows: melting step at 95°C for 1 min, annealing at 58°C for 1 min and elongation at 72°C, for 40 cycles. At the end of the PCR run, a dissociation curve was determined by ramping the temperature of the sample from 60 to 95°C while continuously collecting fluorescence data. Non template controls were included for each primer pair to check for any significant levels of contaminants. Values were normalized by the mean of the 3 reference genes Hypoxanthine-guanine phosphoribosyltransferase, elongation factor 1-a and ribosomal protein S27a.

### Morphological analysis

Eyes, from control (*Crb2F* ^/+^/Chx10Cre^+/-^ and *Crb2* ^*F/F*^) and *Crb2*Chx10 cKO (*Crb2F/F/*Chx10*Cre* ^*+/-*^) mice, were collected at different time points: P10, 1 month-old (1M), 3M (n=3-4/age/group). Eyes were enucleated and fixed at room temperature with 4% paraformaldehyde in PBS for 20 minutes. After fixation, the eyes were dehydrated for 30 minutes in 30%, 50%, 70%, 90% and 96% ethanol and embedded in Technovit 7100 (Kulzer, Wehrheim, Germany), according to the manufacture instructions and sectioned (3 µm). Slides were dried, counterstained with 0.5% toluidine blue and mounted under cover slips using Entellan (Merk, Darmstadt, Germany). Bright field digital images were generated by a Leica epifluorescence microscope (DMRD), using LAS AF v2.4.1 software.

### Immunohistochemical analysis

Eyes from P10 and 2M animals (n=3-6) were enucleated and fixed during 20 min in 4% paraformaldehyde in PBS. Subsequently, the tissues were cryo-protected with 30% sucrose in PBS, embedded in Tissue-Tek O.C.T. Compound (Sakura, Finetek) and used for cryosectioning. Cryosections (7 µm) were rehydrated in PBS. Samples were blocked for 1 h using 10% goat or donkey serum, 0.4% Triton X-100 and 1% bovine serum albumin (BSA) in PBS. The following primary antibodies were used: β-Catenin (1:100; BD Biosciences), Catenin pp120 (P120) (1:100; BD Biosciences), N-Cadherin (1:100; BD Biociences), Calretinin (1:250; Chemicon), APC-conjugated CD11b (1:100; eBioscience), PE-conjugated CD45 (1:100; Emeelca), Cone arrestin (1:500; Millipore), CRB1 (AK2 [7]; 1:100), CRB2 (1:100; Thermo scientific), Glial Fibrillary Acidic protein (GFAP) (1:250; Dako), Glutamine Synthetase (GS) (1:250; BD Biosciences), M-Opsin (1:250; Chemicon), PALS1 (1:1000; Proteintech), PAR3 (1:100; Upstate), PAX6 (1:50; Developmental Studies Hybridoma Bank), PKCα (1:200; BD Transduction Laboratories), Rhodamine Peanut agglutinin (PNA) (1:150; Vector Laboratory), PSD-95 (1:200; Cell Signaling), Recoverin (1:500; Chemicon), Rhodopsin (1:250; Millipore), MPP4 (AK4 [7]; 1:250), MUPP1 (1:200; BD Biosciences), Nectin1 (1:100; MBL), SOX9 (1:250; Millipore), Zona occludens-1 (ZO-1) (1:100; Zymed). The primary antibodies were diluted in 0.3% goat or donkey serum, 0.4% Triton X-100 and 1% BSA in PBS and incubated for 16 h at 4°C. Fluorescent-labeled secondary antibodies were goat anti-mouse or goat anti-rabbit IgGs conjugated to Cy3, Alexa 488 or Alexa 555 (1:500; Jackson Immunoresearch, Stanford, USA and Life Technologies) were diluted in 0.1% goat or donkey serum in PBS and incubated for 1 h at room temperature. Retina sections were counterstained and mounted with Vectashield hard-set mounting medium with DAPI (H1500, Vector Laboratories). Sections were imaged on a Leica SP5 confocal laser scanning microscope (CLSM). Confocal images were processed with Adobe Photoshop CS6 extended v13.0 x64.

### Quantification of Müller glia cells

Müller glia cells were counted at P10 using a SOX9 antibody. Five retina sections from three *Crb2*Chx10 cKO and three control mice were used. Retina sections were counterstained and mounted with Vectashield Hard-Set Mounting Medium with DAPI (H1500, Vector Laboratories). Total number of cells was determined by manually counting of positive cells on digital images generated by a Leica epifluorescence microscope, using LAS AF v2.4.1 software. (n) Represents the number of individual sections.

### Statistical analysis

Normality of the distribution was tested by Kolmogorov-Smirnov test. Statistical analysis by Student’s t test or by Mann Whitney U test in case of a non-normal distribution. Values of *P<0.05, **P<0.01, ***P<0.001 were considered to be statistically significant. Values are expressed as means ± SEM. Calculation were made using the SPSS statistical package version 17.0.

## Results

### Gene expression profile

The CRB2 protein is expressed during retinal development in radial glial progenitor cells, and after differentiation in cone and rod photoreceptors and Müller glia cells [4]. Since these cells span the entire retina, we chose to use whole neural retinas (without the retinal pigment epithelium) in gene expression profile studies. To reduce variation and to standardize the genetic background of *Crb2* cKO mice, the animals were backcrossed from mixed to 99.9% C57BL/6J background. Variation in the genetic background may influence the gene expression, as described before for the *Crb1* knockout (*Crb1* ^*-/-*^) mice [36,42]. Approximately 6 µg of total RNA was extracted from each retina (Table S1 and Table S2). The values of the A260/A280 ratio were in between 1.8 to 2.0 (Table S1 and Table S2), which indicated the absence of contaminating proteins. The A260/A230 ratios were larger than 2.0, which indicated the absence of organic compounds that can interfere with the labelling reaction, such as guanidinium isothiocyanate, alcohol and phenol as well as cellular contaminants such as carbohydrates. The total RNA quality was assessed using the Agilent 2100 Bioanalyser and the respective software, which provides a RNA Integrity Number (RIN) of maximally 10. The samples had on average a RIN of 9.3 indicating high quality of the total RNA.

 We used a whole mouse gene expression microarray that contains over 44,000 probes. To elucidate the molecular events that precede and lead to the morphological phenotype in the *Crb2* retina-specific conditional knockout mice, retinal tissue was collected at postnatal day 0 (P0), P3, P6 as well as P10. Five different retinas from *Crb2*Chx10 cKO (*Crb2F/F/*Chx10*Cre* ^*+/-*^) and the control (*Crb2F* ^/+^/Chx10Cre^+/-^) from each time point were used to cover the onset and first stages of the morphological phenotype observed in the mutant retinas. Equal amounts (825 ng) of Cy3- and Cy5-labelled RNA were hydrolysed and hybridized to a microarray, the hybridization pairs are indicated in Table S3. Five microarrays were processed for each time point. To detect differentially expressed genes a 2-way analysis of variance (ANOVA) using age and genotype as grouping factors was performed. The Benjamini–Hochberg method was used to correct for multiple testing. *P*-values < 0.05 after correction were considered significant. Although we observed substantial changes in gene expression between time points (see section *hierarchical clustering*), our analysis did not reveal significant differences in gene expression between mutant and control retinas. A second analysis, where we used t-tests to compare gene expression between *Crb2* cKO and control retinas at each time point separately, also did not reveal significant changes in gene expression. A description of the top 100 genes with the lowest *P*-values for each time point can be found in the Table S5, Table S6, Table S7 and Table S8.

**Figure 1:**
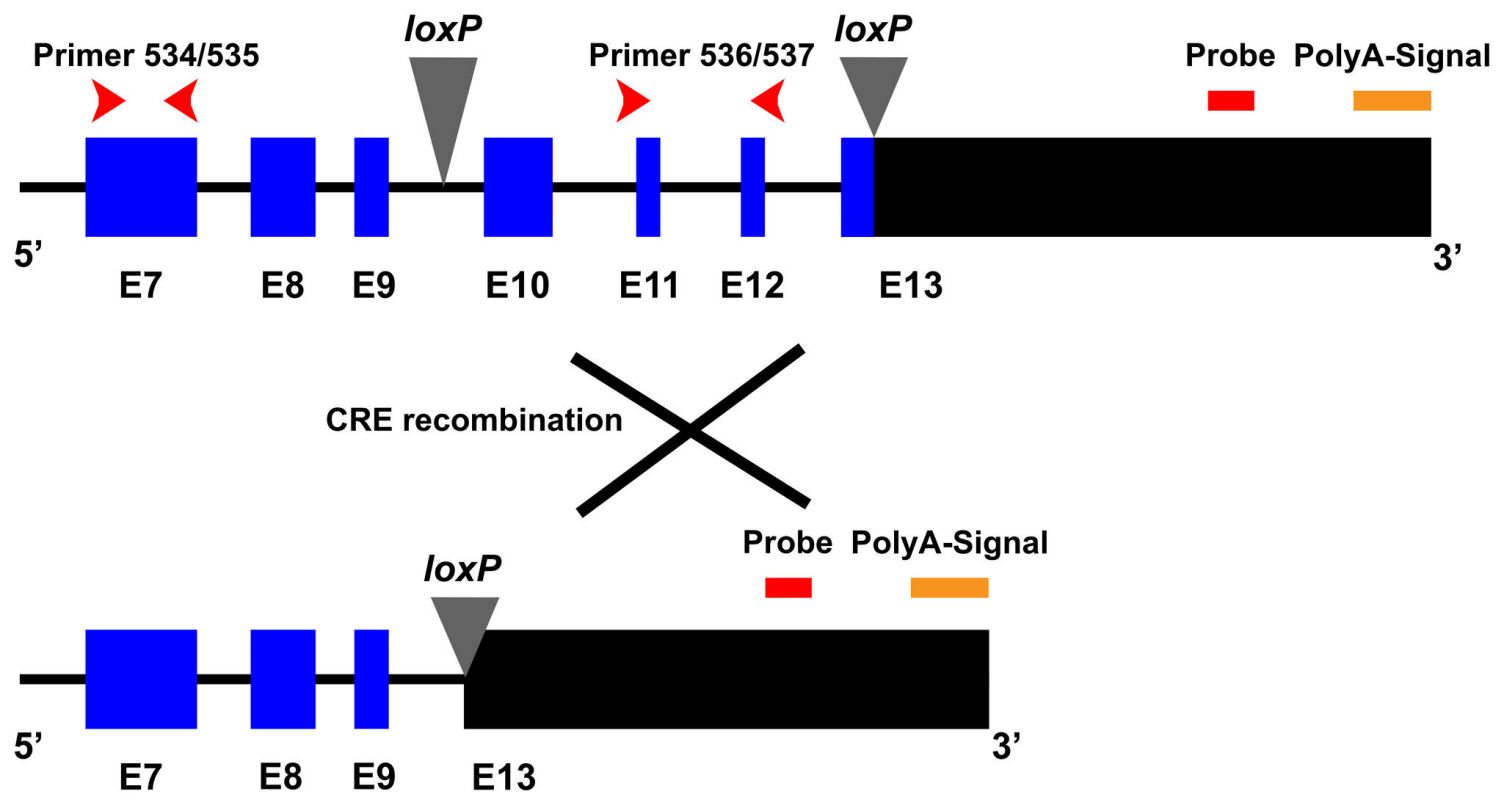
Localization of the *loxP* sites and Crb2 probe in the Crb2 targeting construct. In the Crb2 targeting construct the *loxP* recombination sites are located in intron 9 and within exon 13 in the 3′ non-coding region. Upon Cre-mediated recombination the exons 10, 11, 12 and part of exon 13 are removed. The 60 bp probe for the Crb2 gene is located downstream of the 3’ *loxP*, before the polyadenylation signal. Two sets of primers were used to detect changes in Crb2 expression, they were located in exon 7 (primer 534/535) and in exon 11/12 (primer 536/537). In blue are represented the coding exons of the gene, in black the 3’ untranslated region.

The *Crb2* cKO mice were constructed in such way that the full length CRB2 protein could not be produced in the homozygote mutant retinas. The 5’ *loxP* site was inserted in intron 9, the 3’ *loxP* site was inserted in exon 13 behind the stop codon. The deleted area contains part of the CRB2 extracellular domain, and the entire transmembrane domain and the 37 amino acids of the C-terminal intracellular domain. Cre mediated recombination deleted coding exons 10-13 and resulted in a nonsense mutation with premature truncation of the CRB2 protein at amino acid 871. The unique 60 base pair *Crb2* probe (ID: 235570) in the array is located in between the 3’ *loxP* site and the polyadenylation sequence of the gene (Figure 1). In the microarray study, the *Crb2* transcript was not downregulated in the *Crb2* cKO retinas. 

**Figure 2:**
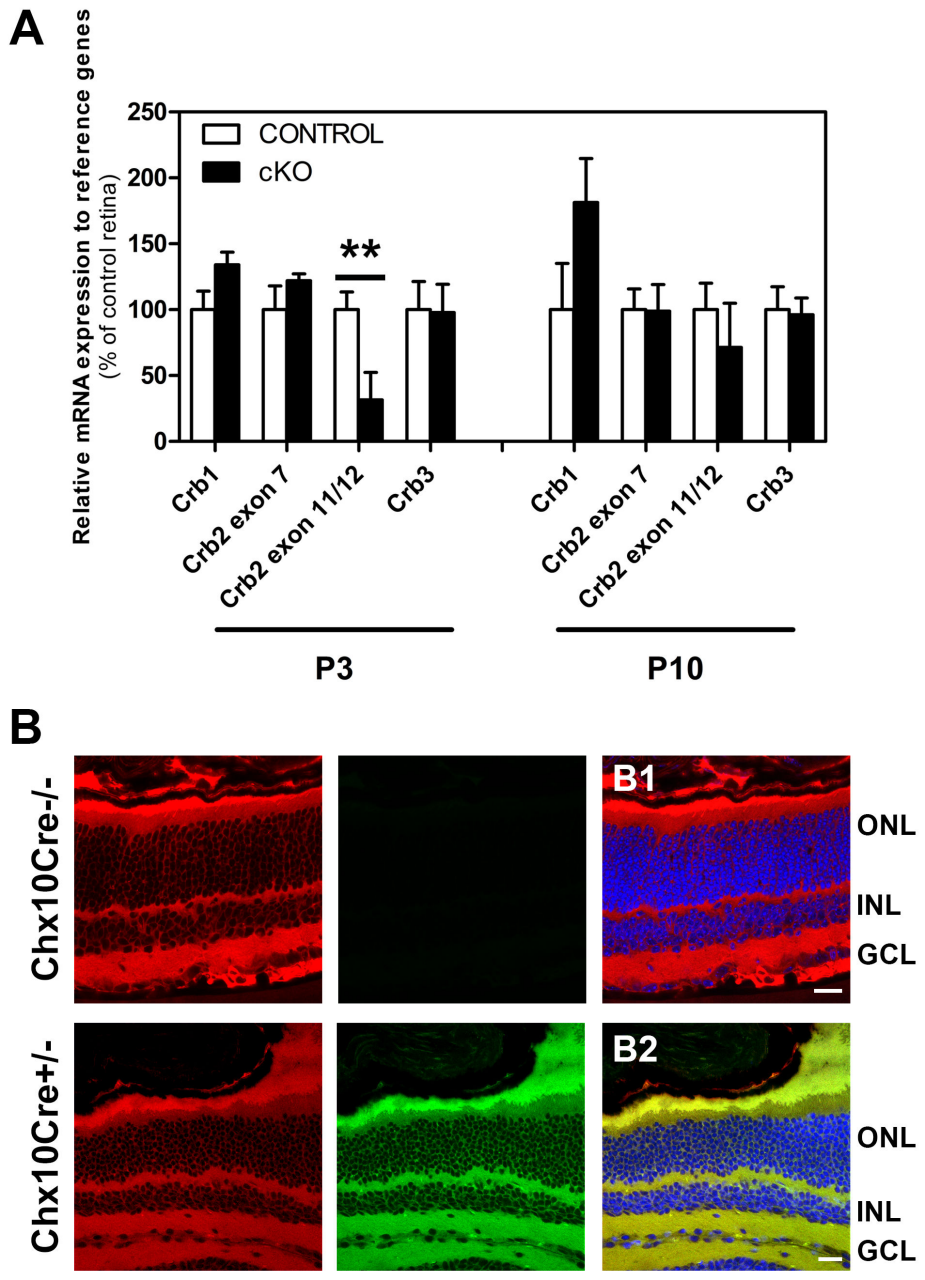
Transcript levels of the Crumbs family members. Transcript levels of Crb2, Crb1 and Crb3 measured by quantitative real time PCR at P3 and P10 (**A**), in 5-6 control and Crb2 cKO retinas. At P3, we detected a decrease of approximately 70% in the Crb2 transcript using primers located between the recombined area (*P* = 0.004). At P10 we did not found differences in the same transcript (*P* = 0.381). No differences were found in the levels of CRB1 (P3: *P* = 0.116; P10: *P* = 0.277) and CRB3 (P3: *P* = 0.912; P10: *P* = 0.869) transcripts. Data are presented as mean ± SEM **P<0.01. Evaluation of the recombination efficiency of a Chx10*Cre* mouse line (**B**). The mT/mG reporter mouse line expresses membrane-targeted red fluorescent protein. After Chx10Cre-mediated recombination, the mT sequence is excised allowing expression of membrane-targeted enhanced green fluorescent protein (mG). Confocal laser scanning microscope pictures of (1M) retina sections from mT/mG (B1) and mT/mG::Chx10Cre (B2). While in the mT/mG only mT signal could be detected, in mT/mG*Chx10Cre retinas expression of mT and mG was found, suggesting mutant adjacent to wild type cells. GCL, ganglion cell layer; INL, inner nuclear membrane; ONL, outer nuclear layer. Scale bars: 20 μm.

To study in more detail the levels of *Crb2* transcript in the *Crb2* cKO retinas we performed quantitative real time - polymerase chain reaction (qRT-PCR) using two different sets of primers, one located outside of the recombined area (exon 7) and another set in the recombined area (exons 11/12) (Figure 1). At P3, we observed a decrease of 70% in the levels of *Crb2* transcript compared to the control, using primers located in the recombined area (between exons 10-13) (*P* = 0.004) (Figure 2A). However, we did not detect changes in the *Crb2* levels using the primers located outside the recombined area (exon 7) (Figure 2A). The data suggest that recombination and deletion of exons 10-13 occurred in the knockout retinas, what suggest again the existence of a truncated transcript. At P10, we did not observe differences in the levels of *Crb2* transcript, using primers located in the recombined area (*P* = 0.381) (Figure 2A), what suggest that the proportion of *Crb2* null cells is decreased compared to non-recombined cells, which might be due to increased apoptosis of *Crb2* mutant photoreceptors [4].

We previously showed using an antibody against the intracellular C-terminal 37 amino acids domain of CRB2 that the protein was removed from the outer limiting membrane from embryonic day 12.5 onwards [4]. So, the truncated transcript is recognized by the probe on the microarray, and expression of the truncated transcript is not changed compared to the full length transcript.

These findings may be explained in part by the mosaic genetic nature of the mutant retinas which contain mutant next to wild type cells due to insufficient levels of *Cre* expression in a subset of cells. To evaluate the mosaicism of the Chx10*Cre* mouse line we crossed the Chx10*Cre* transgenic mouse line with a *mT/mG* (membrane-targeted tandem-dimer-Tomato RFP / membrane-targeted Enhanced GFP) reporter mouse line [37]. This reporter mouse line expresses red fluorescent protein before and green fluorescent protein after Cre-mediated recombination. While in the *mT/mG* retinas only red fluorescent protein could be detected (Figure 2B1), in *mT/mG::*Chx10*Cre* (heterozygous *mT/mG*, heterozygous Chx10*Cre*) retinas expression of red and green fluorescent proteins was found (Figure 2B2), suggesting the presence of mutant adjacent to wild type cells.

In mammals, the *Crb* gene family is composed of *Crb1*, *Crb2* and *Crb3*, the lack of changes in the transcriptome after loss of *Crb2* gene function might be due to compensation by *Crb1* or *Crb3*. To test this, we used qRT-PCR to determine the levels of the *Crb1* and *Crb3* transcripts, but whereas we detected a decrease in *Crb2* transcripts, we did not observe changes in the levels of *Crb1* or *Crb3* transcripts between control and knockout retinas (Figure 2A).

### Crb2 and signalling pathways

**Figure 3:**
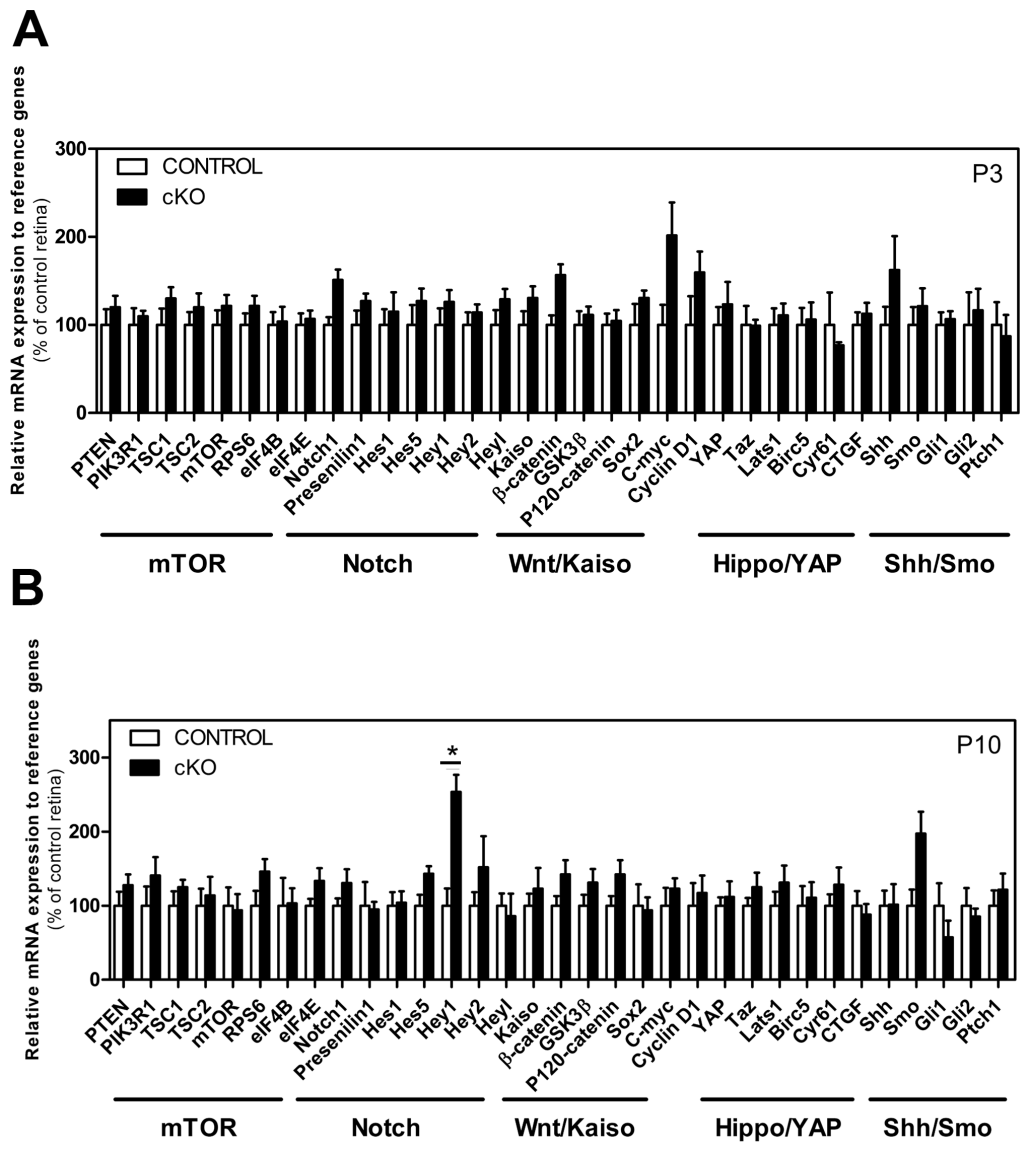
Crb2 and signalling pathways. Transcript levels measured by quantitative real time PCR at P3 (A) and P10 (B) in 5-6 control and Crb2 cKO retinas showed only changes in Hey1 (*P* = 0.041). P3: *Notch1* *P* = 0.144, *Kaiso* *P* = 0.440, β-catenin *P* = 0.082, c-myc *P* = 0.711, cyclin D1 *P* = 0.226, Cyr61 *P* = 0.4687, *Shh* *P* = 0.411. P10: *Smo* *P* = 0.182, *gli1* *P* = 0.262, *P120-catenin* *P* = 0.228, β-catenin *P* = 0.837, Hey2 *P* = 0.472, *elF4E* *P* = 0.236, RPS6 *P* = 0.196, *PIK3K1* *P* = 0.375. Data are presented as mean ± SEM *P<0.05; **P<0.01.

We investigated by qRT-PCR the role of *Crb2* in the regulation of Notch1, mTORC1, Hippo, Wnt and sonic hedgehog pathways in *Crb2* cKO retinas at P3 and P10. We did not observe major changes in these pathways (Figure 3). Only the *Hey1* transcript was significantly changed at P10, with an increase of nearly 2.5-fold in the knockout retinas compared to the control (*P* = 0.041). The qPCR data confirmed the data obtained by microarray analysis. 

### Hierarchical clustering

**Figure 4:**
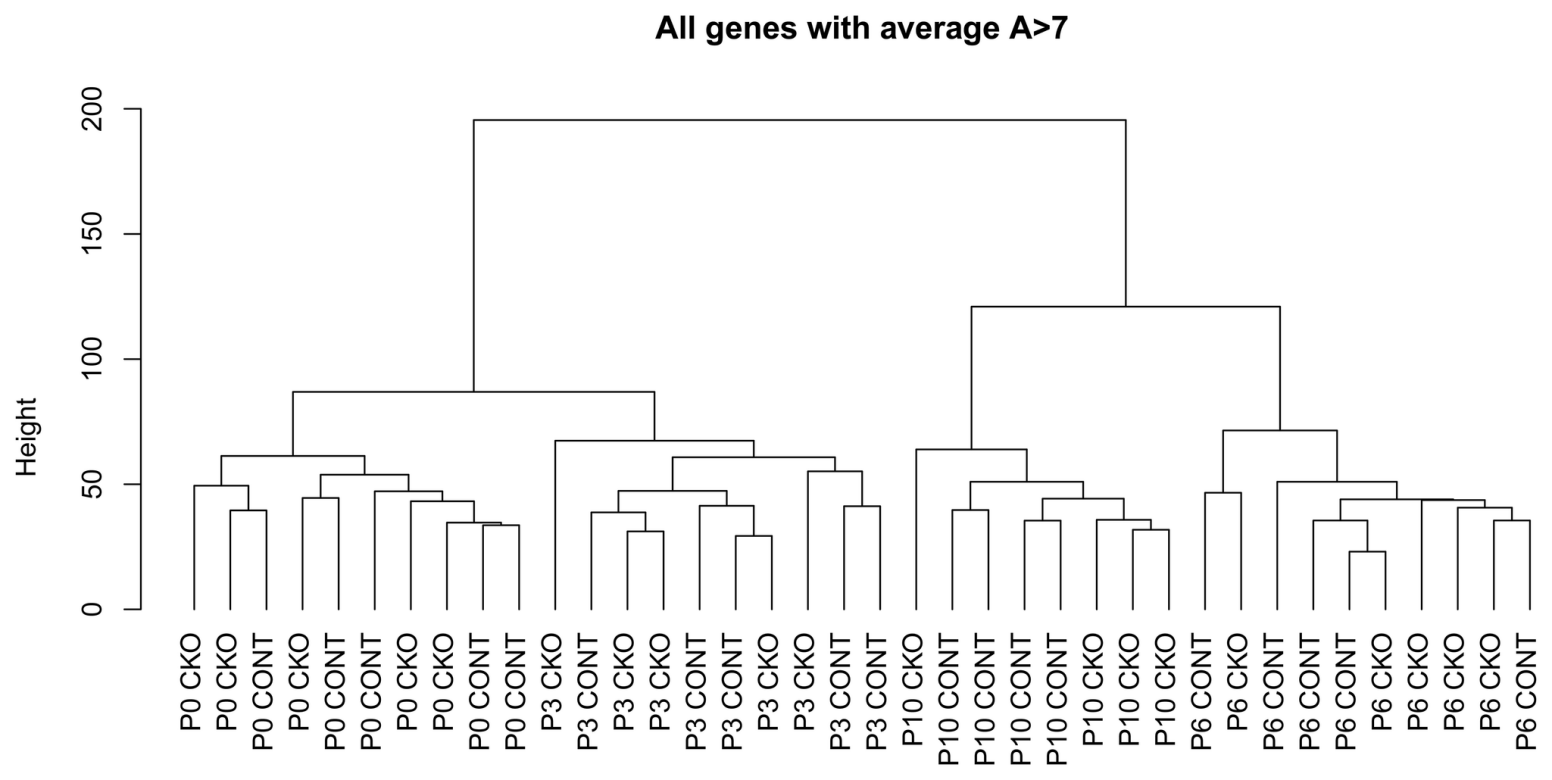
Tree-chart of correlative gene expression patterns from gene cluster analysis.

Analysis of cluster gene expression was performed at distinct retinal development time points. Figure 4 and Figure S1 show the correlative gene expression from P0 to P10. Two phases are visible, P0-P3 and P6-P10 based on their correlative distance of gene expression. This is in accordance with the findings described by Zhang et al, 2006. They described two phases as a "developmental phase" (from E12.5 to P5) and a "functional phase" (from P7 on) [24]. This analysis also revealed that the gene expression variation induced by differences in development time points was much larger that the differences between *Crb2* cKO and control retinas.

### The retinal phenotype of Crb2 cKO on C57BL/6J background

The phenotype of retinas lacking CRB2 from early retinal development was previously described [4]. Those animals were kept on a mixed genetic background (50% OLA129 and 50% C57BL/6J). In order to reduce variation and to standardize the genetic background of these mice, the animals were backcrossed to 99.9% C57BL/6J background. Variation in the genetic background may influence the severity of the retinal phenotype, as described before for the *Crb1* knockout (*Crb1* ^*-/-*^) mice [7,22]. So, to study the effect of the genetic background on the morphological phenotype of *Crb2* null retinas, retinas of P10, and 1- and 3 months-of-age were analysed.

**Figure 5:**
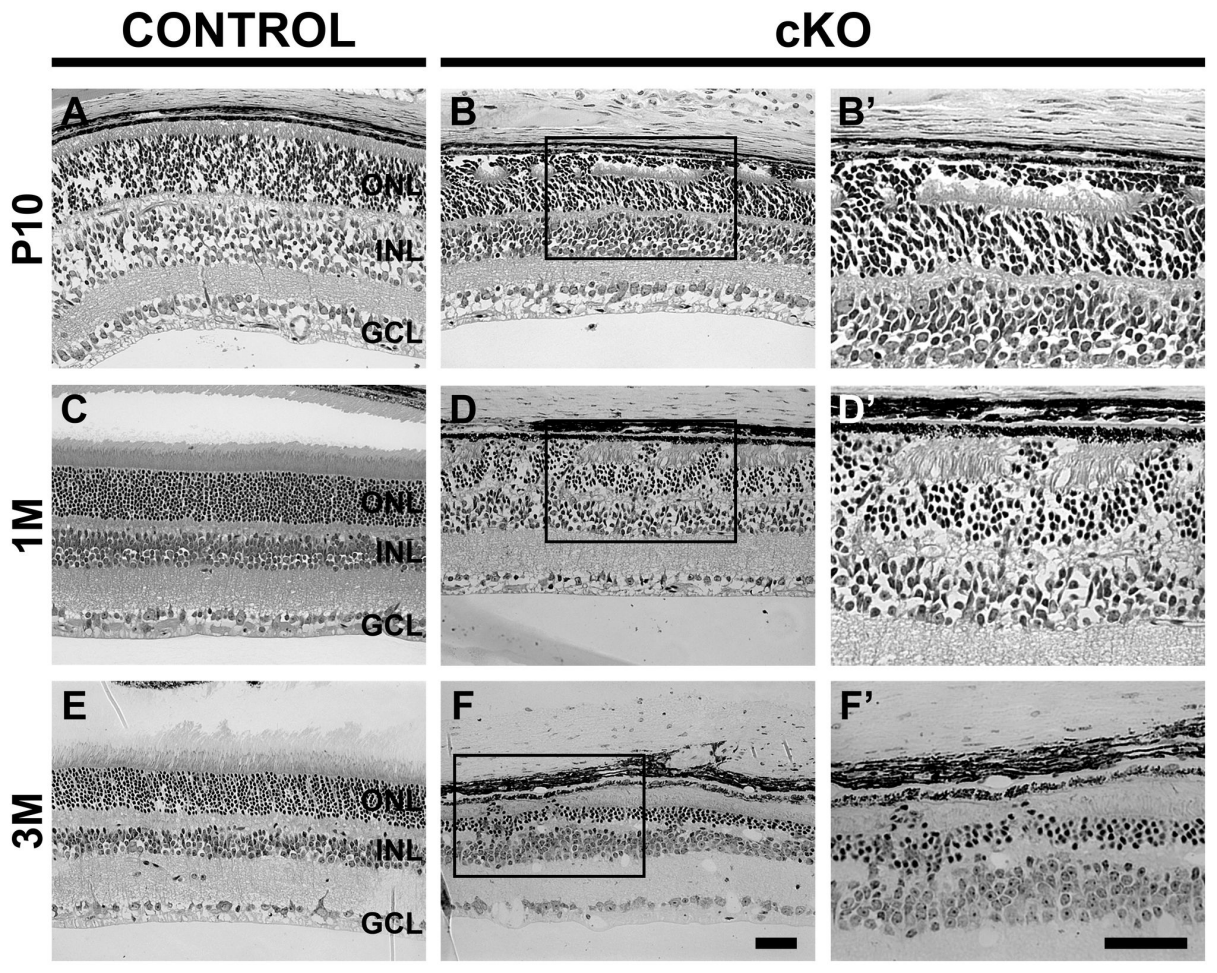
Loss of CRB2 results in retinal disorganization in mice on C57BL/6J genetic background. Toluidine stained light microscopic pictures, of retina sections, from the control (**A**, **C** and **E**) and from the *Crb2*Chx10 cKO on C57BL/6J genetic background (**C**, **D** and **F**), at different ages, P10 - (**A**, **B**), 1M - (C, D), 3M - (**E**, **F**). At P10 (**B**), several photoreceptor nuclei were localized ectopically in the subretinal space. At 1M (**D**) protrusion of photoreceptor cell nuclei in the subretinal space and gaps in the outer limiting membrane were observed. At 3M (**F**) we observed thinner outer nuclear layers, with rows of photoreceptors cells protruding into the subretinal space through the outer limiting membrane and protrusions of inner nuclear layer cells into the outer nuclear layer. No abnormalities were observed in the control. GCL, ganglion cell layer; INL, inner nuclear layer; ONL, outer nuclear layer. Scale bar: 50 µm.

 The phenotype of retinas lacking CRB2 on 99.9% C57BL/6J genetic background appeared grossly the same as on 50% C57BL/6J genetic background [4] at the time points analysed (Figure 5 and Figure S2). At P10, disruptions of the outer limiting membrane and ectopic photoreceptor nuclei near to the retinal pigmented epithelium were observed (Figure 5B and Figure S2A).

 Adult retinas on 99.9% or 50% C57BL/6J genetic background showed similar progression of the morphological phenotype. In 1 month-old retinas, protrusions of photoreceptors into the subretinal space were observed (Figure 5D and Figure S2B). Three month-old retinas also showed protrusion of photoreceptor nuclei into the subretinal space, in these retinas the retinal thickness decreased to only a few (4-6) photoreceptor nuclei in a row, the outer plexiform layer was thinner and protrusion of nuclei from the inner nuclear layer into the outer nuclear layer were found (Figure 5F and Figure S2C).

 In conclusion, the CRB2 null retinas on 99.9% C57BL6/J background showed a similar progression of the phenotype than on 50% C57BL6/J background.

### Early morphological retinal phenotype of the Crb2 cKO on C57BL/6J background

**Figure 6:**
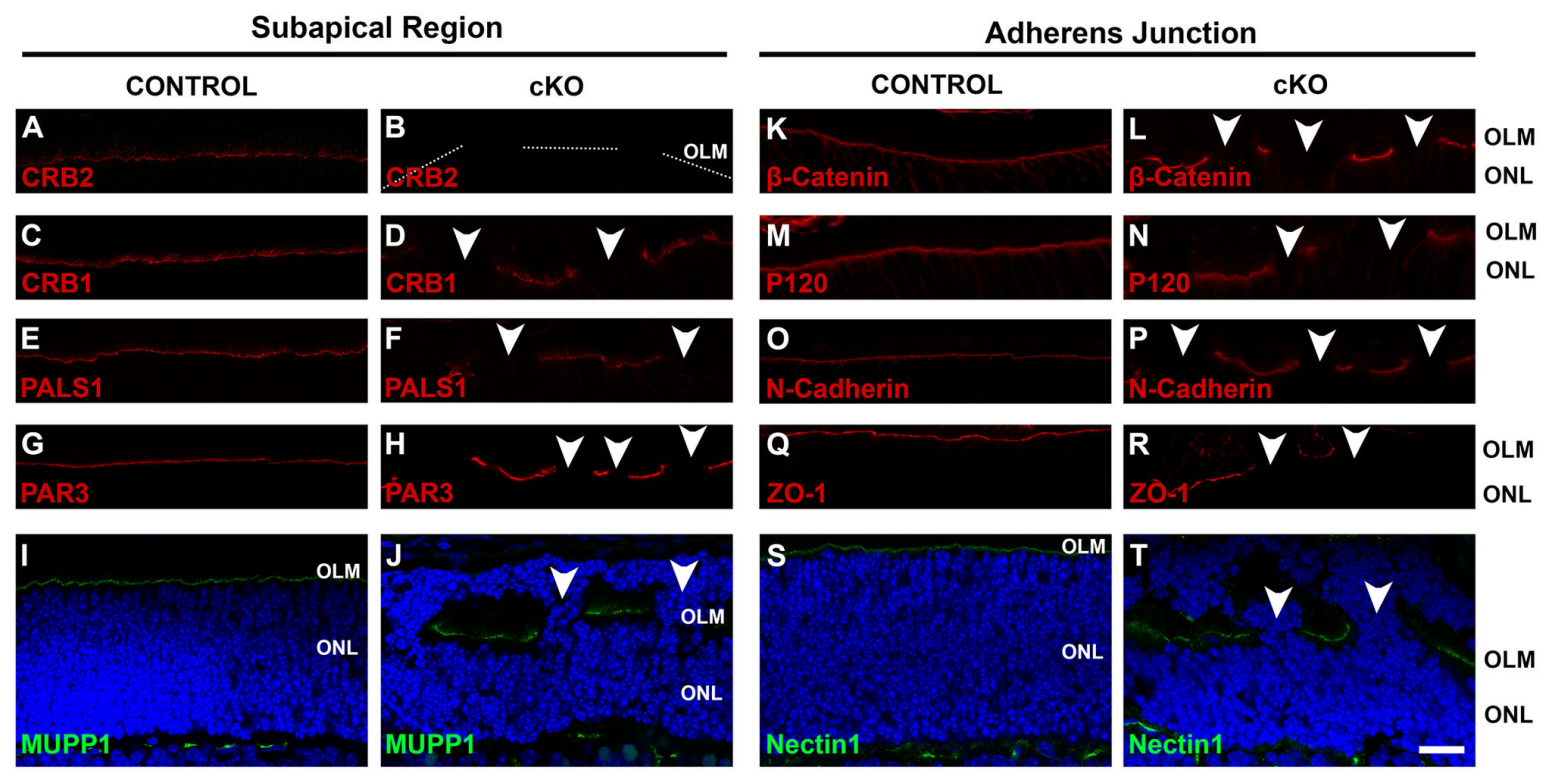
Lack of CRB2 leads to the disruption of the apical CRB protein complex. Immunohistochemistry pictures from (P10) retina sections stained for subapical region markers: CRB2 (**A**, **B**), CRB1 (**C**, **D**), PALS1 (**E**, **F**), PAR3 (**G**, **H**), MUPP1 (**I**, **J**) and for adherens junction markers: β-Catenin (**K**, **L**), Catenin pp120 (P120) (**M**, **N**), N-Cadherin (**O**, **P**), Zona occludens-1 (ZO-1) (**Q**, **R**), Nectin1 (**S**, **T**). CRB2 was absent in the knockout retina (**B**), in contrast to control (**A**). CRB1, PALS1 and MUPP1 staining showed disruption of the CRB complex at the subapical region at sites of cellular mislocalization (**D**, **F** and **J**). PAR3 was also lost at sites of disruption (**H**). Staining using adherens junctions markers β-Catenin (**L**), P120 (**M**), N-cadherin (**P**), ZO-1 (**R**) and Nectin1 (**T**) showed disruption of the adherens junctions. Moreover, ectopic photoreceptor nuclei protruded into the subretinal space (**J** and **T**). No morphological changes were observed in the control retinas. OLM, outer limiting membrane; ONL, outer nuclear layer. Scale bars: 20 μm.

As in CRB2 null retinas on mixed background, the CRB2 null retinas on 99.9% C57BL6/J background showed at postnatal day 10 a consistent disorganized morphology with many photoreceptors misplaced in the subretinal space adjacent to the retinal pigmented epithelium, thus we examined 10-days old retinae in detail using immunohistochemistry. At P10, the outer limiting membrane was disrupted throughout the mutant retina, and although CRB2 was lost in the entire retina (Figure 6B), loss of the CRB complex proteins, CRB1 (Figure 6D), PALS1 (Figure 6F), MUPP1 (Figure 6J) and of the PAR complex member PAR3 (Figure 6H) occurred only at sites of protrusions of photoreceptor nuclei into the subretinal space. Similar disruptions of adherens junction markers, β-Catenin (Figure 6L), Catenin pp120 (Figure 6N), N-Cadherin (Figure 6P), ZO-1 (Figure 6R) and Nectin1 (Figure 6T) were observed. 

**Figure 7:**
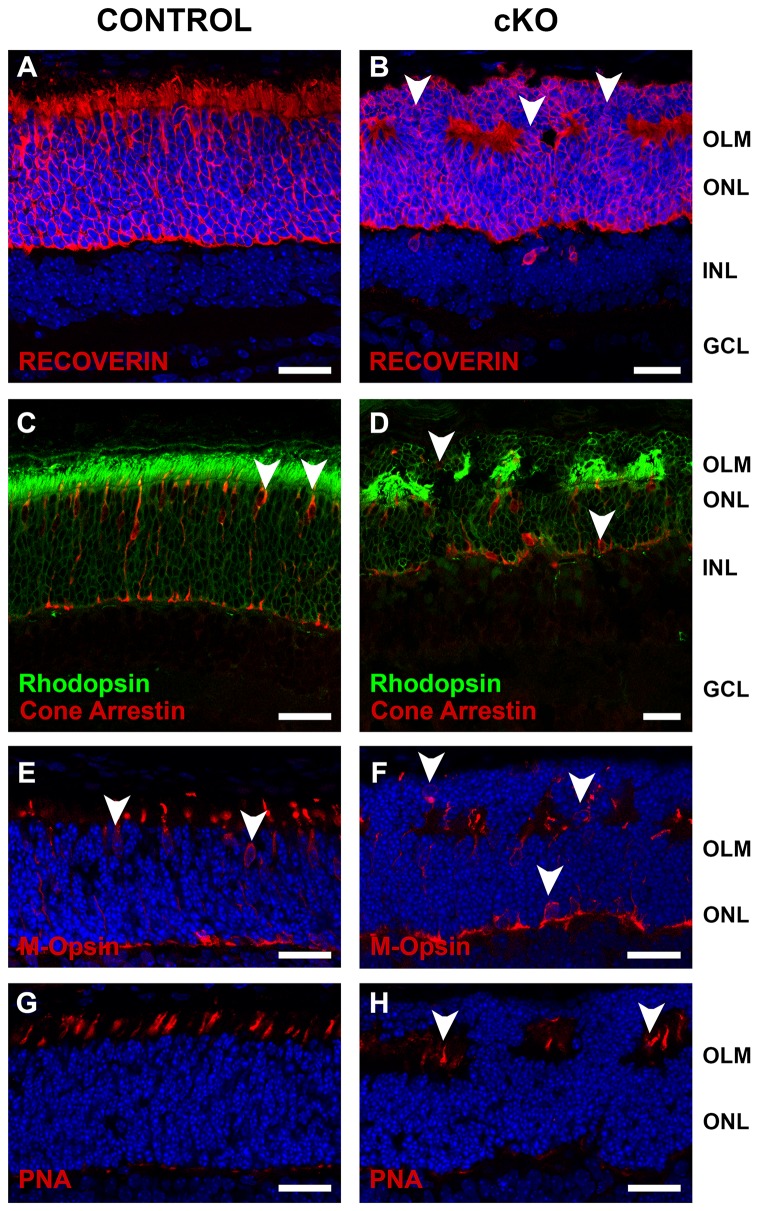
Loss of CRB2 affects lamination of photoreceptor cells. Immunohistochemistry pictures from 10 days old mouse retinas. Sections were stained with antibodies against: recoverin (**A**, **B**), rhodopsin and cone arrestin (**C**, **D**), M-opsin (**E**, **F**), peanut agglutinin (PNA) (**G**, **H**). In the mutant retinas several photoreceptor nuclei localized in the subretinal space were positive for recoverin (**B**) and rhodopsin (**D**). Some cone arrestin (**D**), M-opsin (**F**) positive nuclei were also misplaced in the subretinal place, showing that also cone photoreceptors lamination was affected. Outer segment from the cone photoreceptor cells, stained with PNA, were present in both retinas (**G**, **H**). However, in the mutant retinas these segments were located between photoreceptor nuclei and not in contact with the retinal pigmented epithelium (**H**). GCL, ganglion cell layer; INL, inner nuclear layer; OLM, outer limiting membrane; ONL, outer nuclear layer. Scale bars: 25 µm.

 In CRB2 null retinas several ectopic photoreceptor nuclei were present in the subretinal space immediately adjacent to the retinal pigment epithelium (Figure 5C, Figure 6J, T and Figure 7B, D, F, H). Most of these cell bodies were positive for recoverin (Figure 7B) and rhodopsin (Figure 7D). The lamination of cone photoreceptor cells, stained by cone arrestin (Figure 7D), M-opsin (Figure 7F) was also affected. Many cone nuclei were misplaced in the subretinal space. Some of the misplaced photoreceptors showed polarity defects with a reverse apical-basal orientation. The cone outer segments were present in both retinas (Figure 7G, H), however in the mutant retinas the outer segments were located between the correct localized outer nuclear layer and the ectopic photoreceptors nuclei in the subretinal space (Figure 7H), instead of been located immediately adjacent to the retinal pigmented epithelium.

**Figure 8:**
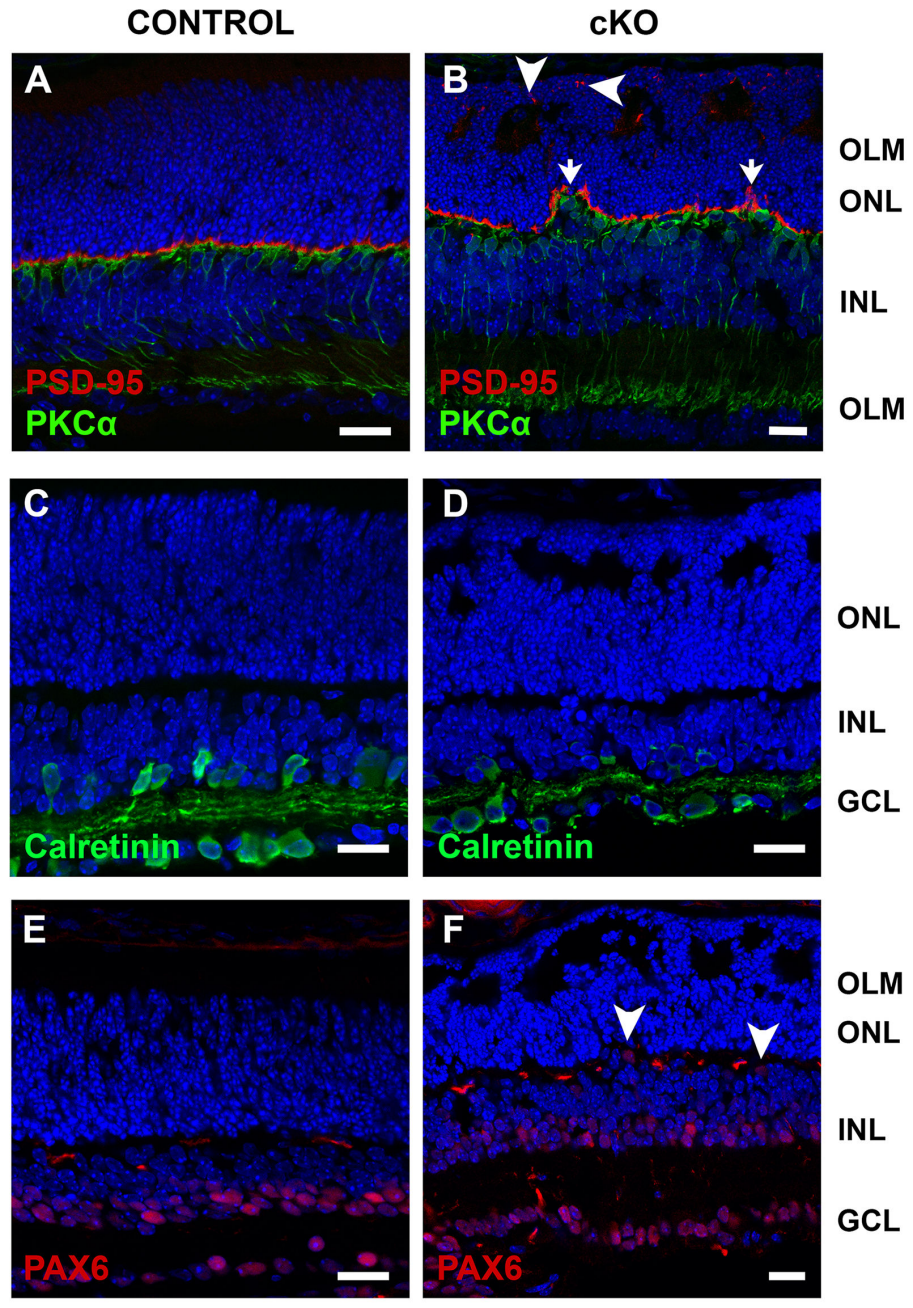
Retinas lacking CRB2 show ectopic synapses. Immunohistochemistry pictures from P10 mouse retinae. Retina sections were stained with antibodies against: PKCα and PSD-95 (**A**, **B**), Calretinin (**C**, **D**) and PAX6 (**E**, **F**). In the *Crb2*Chx10 cKO retinae the outer plexiform layer, stained by an PSD-95 antibody, is thinner and showed some disruptions (arrows), ectopic synapses were also detected in the outer nuclear layer (arrowheads) (**B**). Some bipolar cells, stained with anti-PKCα (**B**), and some PAX6-positive amacrine cells (**F**) were misplaced in the outer plexiform layer of the mutant retinas. Calretinin positive amacrine cells (**D**) seemed not affected in the *Crb2*Chx10 cKO retinae. No morphological changes were observed in the control retinae. GCL, ganglion cell layer; INL, inner nuclear layer; OLM, outer limiting membrane; ONL, outer nuclear layer. Scale bars: 20 µm.

 Some of the ectopic photoreceptor cells were not able to establish synapses in the outer plexiform layer, demonstrated by ectopic localization of PSD-95 (Figure 8B) and MPP4 (data not shown). 

 Most of bipolar cells stained by anti-PKCα (Figure 8B), amacrine calretinin-positive cells (Figure 8D), and amacrine and ganglion cells (PAX6-positive, Figure 8F) were correctly localized, showing no major defects in the lamination of these cell types, however in some areas some misplaced bipolar (Figure 8B) and amacrine cells (Figure 8F, arrowheads) were observed in the outer plexiform layer. 

**Figure 9:**
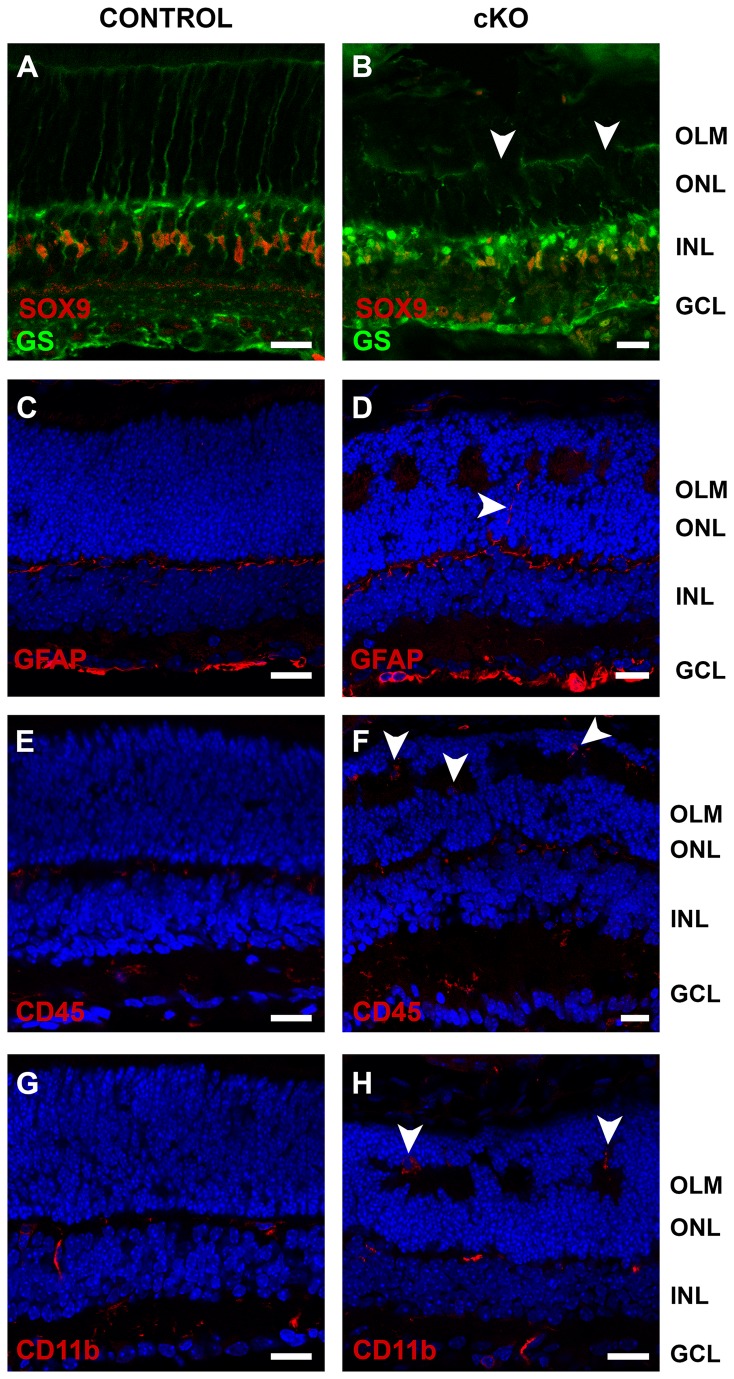
Loss of CRB2 results in gliosis and microglia activation. Immunohistochemistry pictures from P10 mouse retinae. Sections were stained with antibodies against: SOX9 and Glutamine synthetase (GS) (**A**, **B**), GFAP (**C**, **D**), CD45 (**E**, **F**), CD11b (**G**, **H**). The location of nuclei of Müller glia cells, stained with SOX9 was not altered in the mutant retinas (**B**), however disruption at the apical end feet of the Müller glia cells were observed at sites with photoreceptor protrusions (**B**). The mutant retinas showed activated Müller glia cells, detected by a moderate increase in the GFAP staining in the outer nuclear layer (arrowhead) (**D**). An increase in activated microglia cells in the outer nuclear layer, stained with anti-CD45 and anti-CD11b, was detected (**F** and **H**). No morphological changes were observed in the control retinae. GCL, ganglion cell layer; INL, inner nuclear layer; IPL, inner plexiform layer; OLM, outer limiting membrane; ONL, outer nuclear layer; OPL, outer plexiform layer. Scale bars: 20 µm.

 Also, no misplaced SOX9-positive Müller glia nuclei were observed in the CRB2 null retinas (Figure 9B), however in the mutant retinas disruptions at the apical end feet of the Müller glia cell were observed (Figure 9B, arrowheads), these disruptions coincide with the photoreceptor protrusions, suggesting lack of adhesion between Müller and photoreceptor cells. A mild increase in glial fibrillary acidic protein (GFAP) was observed in the outer nuclear layer of the knockout (Figure 9D) compared to the control retinas (Figure 9C), suggesting Müller glia activation.

 As in CRB2 null retinas on mixed background, the *Crb2*Chx10 cKO retinas (99.9% C57BL/6J) showed an increase in the number of Müller glia cells (Control: 15.1±0.4 versus cKO: 17.4±0.7 SOX9 positive cells/100μm ±SEM, *P* = 0.0066, n = 15). In C*rb2*Chx10 cKO retina, we observed ectopic protein expression of two microglia cell markers, CD45 and CD11b, in the misplaced photoreceptor cell layer (Figure 9F, H). 

**Figure 10:**
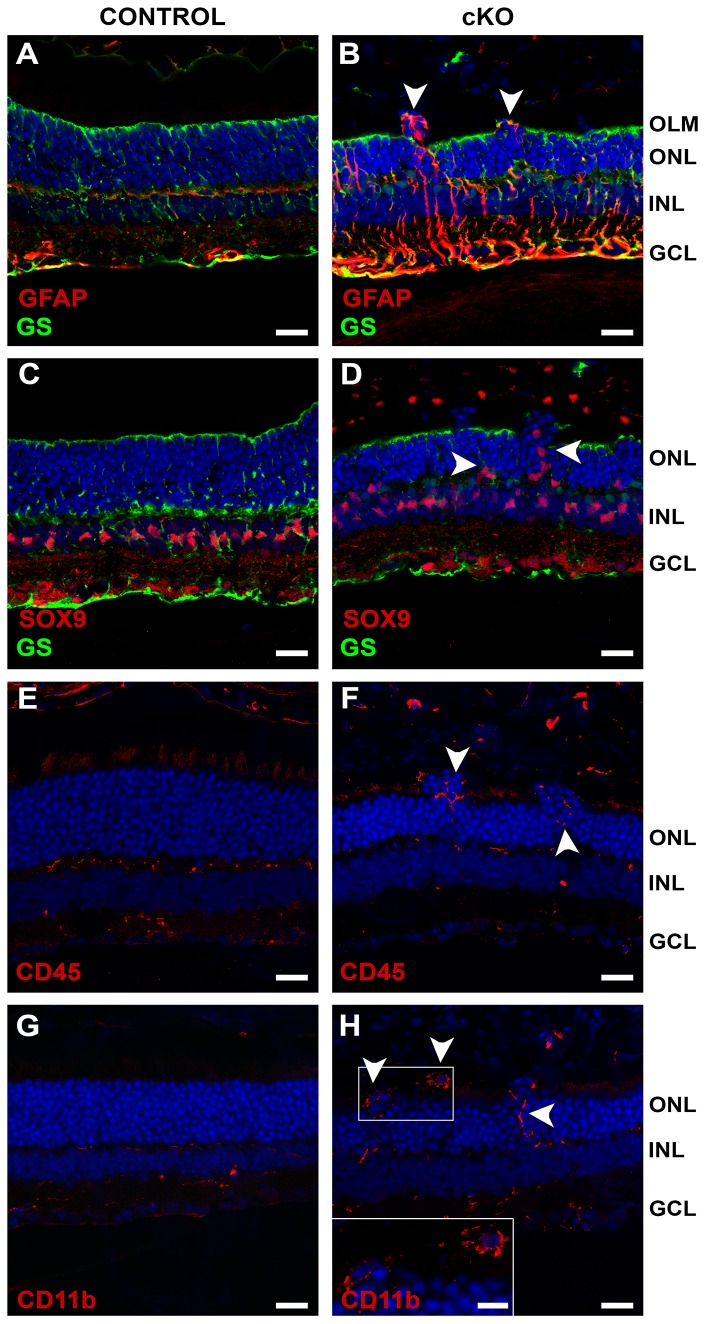
Loss of CRB2 results in gliosis and microglia activation in the adult retina. Immunohistochemistry pictures from 2M mouse retinae. Sections were stained with antibodies against: Glial fibrillary acidic protein (GFAP) and Glutamine synthetase (GS) (**A**, **B**), SOX9 and GS (**C**, **D**), CD45 (**E**, **F**), CD11b (**G**, **H**). The mutant retinas showed activated Müller glia cells, detected by an increase in the GFAP staining in the outer nuclear layer (arrowhead) mainly in areas of cellular mislocalization (**B**). At foci, Müller glia cell nuclei (SOX9-positive) were detected in the outer nuclear layer (arrowheads) (**D**). Staining by anti-CD45 and anti-CD11b showed an increase in ectopic activated microglia cells in the outer nuclear layer and adjacent to ectopic photoreceptor nuclei located in the subretinal space (arrowheads; F and H). No morphological changes were observed in the control retinae. GCL, ganglion cell layer; INL, inner nuclear layer; ONL, outer nuclear layer. Scale bars: 20 µm. Scale bar in the inset: 10 µm.

Gliosis was more evident in 2-months-old mutant retinas, where a prominent increase of GFAP was observed (Figure 10B, arrowheads), mainly at foci of cellular mislocalization. At these sites, ectopic SOX9-positive Müller glia nuclei could be detected in the outer nuclear layer (Figure 10D, arrowheads). Similar as in P10 retinas, an increase in CD45 and CD11b was observed in 2-month-old retinas, these proteins were detected in the outer nuclear layer and adjacent to ectopic photoreceptor nuclei in the subretinal space (Figure 10F, H).

 In conclusion, the absence of differences in transcriptomes between control and CRB2 null retinas is not due to suppression of the CRB2 retinal phenotype on C57BL/6J background.

## Discussion

At the time points of retinal development analysed, the retinas lacking CRB2 showed discrete but consistent phenotypes mainly at the periphery of the retina, with the exception of the P10 time point where the phenotype became severe with the accumulation of misplaced photoreceptor cells in the subretinal space throughout the entire retina. However, no differences were found in the mutant retinas in our gene profiling studies, with exception of *Hey1* at P10. This finding was unexpected since several studies demonstrated that the CRB complex members are able to regulate several important signalling pathways including the Notch1 [9-11], mTORC1 [12,13] and the Hippo pathway [3,14-17]. Moreover, the knockout retinas showed alteration in several cellular parameters, as for example, increase in apoptosis, number of retinal progenitor cells and/or in the number of late born cells such as Müller glia and rod photoreceptor cells. The mislocalized photoreceptors showed an immature morphology and polarity defects. The findings may be explained in part by the mosaic genetic nature of the mutant retinas which contain mutant next to wild type cells due to insufficient levels of *Cre* expression in a subset of cells. The use of a Cre mouse line that expresses Cre in all retinal progenitor cells might circumvent this problem. In the current study, retinas with loss of CRB2 in a mix of mutant radial glial progenitor cells, rod and cone photoreceptors, and Müller glia cells were analysed in the gene expression studies. In future studies, it might be worthwhile to ablate expression of CRB2 by different Cre-drivers specifically in photoreceptors and/or Müller glia cells and perform gene expression profiles on purified photoreceptors and/or Müller glia cells purified by cell sorting.

In previous studies using *Crb1* mutant mice on mixed genetic background (50% OLA129 and 50% C57BL/6J), only a limited number of ﬁve genes showed changes in gene expression in 3-months-old white-light-exposed *Crb1* ^*-/-*^ mice [36]. Three of these genes are involved in inhibition of chromatid separation (*Pttg1*), chromatid cohesion (*Esco1*), or chromosome stabilization (*similar to histone H2b*). Interestingly, downregulation of *Pttg1* was also detected in white light exposed *Crb1* ^*C249W/-*^ retinas that expressed a CRB1 protein with an amino acid substitution in the extracellular domain [42].

 In *Crb2* null retinas, the CRB2 protein is lost from the entire subapical region. However, loss of CRB2 leads to loss of localization of other apical proteins, as for example members of the CRB, PAR and adherens junction complexes, only at foci where mislocalized cells were found.

In conclusion, the results suggest that loss of CRB2 in the developing retina had no major influence on the gene expression profile. 

## Supporting Information

Figure S1 **Principal component analysis (PCA) of all probes and all samples.** PCA analysis separates samples on the age. Each data point/shape represents a sample, with all the arrays presented on the PCA plot. Crb2 conditional knockout (CKO) (red), and control (CONT) (blue) samples, are distinguished by color, and age P0 (piramide), P32 (tetrahedron), P6 (octahedron), and P10 (cube) are distinguished by shape. Given PC 1 describes the largest amount of data variance (34%), the aggregation of the samples is mostly accounted for the sample age. The percentage values in parentheses indicate the proportion of total variance described by each PC. PC 1 principal component 1 (X-axis); PC 2 principal component 2 (Y-axis); PC 3 principal component 3 (Z-axis).
(TIF)

Figure S2 **Loss of CRB2 results retinal disorganization in mice C57BL/6J genetic background.** Toluidine stained light microscopic pictures of retina sections at different ages, P10 - (**A**), 1M - (**C**), 3M - (**D**). Whole section figures, from the CRB2 null retinas, were mounted from individual pictures using stitching/MosaicJ pluging from ImageJA v1.45b. GCL, ganglion cell layer; INL, inner nuclear layer; ONL, outer nuclear layer. Scale bar: 50 µm.
(TIF)

Table S1 **Concentration and integrity of the RNA and cRNA samples used in the microarray (P0 and P3).**
(DOCX)

Table S2 **Concentration and integrity of the RNA and cRNA samples used in the microarray (P6 and P10).**
(DOCX)

Table S3 **Description of the hybridization pairs and distribution of the samples in the arrays.**
(DOCX)

Table S4 **qRT-PCR primers used in this study.**
(DOCX)

Table S5 **Differential gene expression between control and knockout neuroretinas in fold differences, at postnatal day 0.** Top 100 genes ranked on their *P* value given by the students’ t-test (P value) before applying Benjamini–Hochberg (P value bh) method for correct to multiple testing. The expression value to the individual genes for control (CONT) and knockout (CKO) groups (log_2_ intensity), and the fold differences between control and knockout (FC) are also described in the table.
(DOCX)

Table S6 **Differential gene expression between control and knockout neuroretinas in fold differences, at postnatal day 3.** Top 100 genes ranked on their *P* value given by the students’ t-test (P value) before applying Benjamini–Hochberg (P value bh) method for correct to multiple testing. The expression value to the individual genes for control (CONT) and knockout (CKO) groups (log_2_ intensity), and the fold differences between control and knockout (FC) are also described in the table.
(DOCX)

Table S7 **Differential gene expression between control and knockout neuroretinas in fold differences, at postnatal day 6.** Top 100 genes ranked on their *P* value given by the students’ t-test (P value) before applying Benjamini–Hochberg (P value bh) method for correct to multiple testing. The expression value to the individual genes for control (CONT) and knockout (CKO) groups (log_2_ intensity), and the fold differences between control and knockout (FC) are also described in the table.
(DOCX)

Table S8 **Differential gene expression between control and knockout neuroretinas in fold differences, at postnatal day 10.** Top 100 genes ranked on their *P* value given by the students’ t-test (P value) before applying Benjamini–Hochberg (P value bh) method for correct to multiple testing. The expression value to the individual genes for control (CONT) and knockout (CKO) groups (log_2_ intensity), and the fold differences between control and knockout (FC) are also described in the table.
(DOCX)

## References

[1] Young RW (1985) Cell differentiation in the retina of the mouse. Anat Rec 212: 199-205. doi:10.1002/ar.1092120215. PubMed: 3842042. 3842042

[2] Livesey FJ, Cepko CL (2001) Vertebrate neural cell-fate determination: lessons from the retina. Nat Rev Neurosci 2: 109-118. doi:10.1038/35053522. PubMed: 11252990. 11252990

[3] Martin-Belmonte F, Perez-Moreno M (2012) Epithelial cell polarity, stem cells and cancer. Nat Rev Cancer 12: 23-38. PubMed: 22169974. 10.1038/nrc3169 22169974

[4] Alves CH, Sanz Sanz A, Park B, Pellissier LP, Tanimoto N et al. (2013) Loss of CRB2 in the mouse retina mimics human retinitis pigmentosa due to mutations in the CRB1 gene. Hum Mol Genet 22: 35-50. doi:10.1093/hmg/dds398. PubMed: 23001562. 23001562

[5] Park B, Alves CH, Lundvig DM, Tanimoto N, Beck SC et al. (2011) PALS1 Is Essential for Retinal Pigment Epithelium Structure and Neural Retina Stratification. J Neurosci 31: 17230-17241. doi:10.1523/JNEUROSCI.4430-11.2011. PubMed: 22114289. 22114289 PMC6623860

[6] Cho SH, Kim JY, Simons DL, Song JY, Le JH et al. (2012) Genetic ablation of Pals1 in retinal progenitor cells models the retinal pathology of Leber congenital amaurosis. Hum Mol Genet 21: 2663-2676. doi:10.1093/hmg/dds091. PubMed: 22398208. 22398208 PMC3363335

[7] van de Pavert SA, Kantardzhieva A, Malysheva A, Meuleman J, Versteeg I et al. (2004) Crumbs homologue 1 is required for maintenance of photoreceptor cell polarization and adhesion during light exposure. J Cell Sci 117: 4169-4177. doi:10.1242/jcs.01301. PubMed: 15316081. 15316081

[8] Fu X, Sun H, Klein WH, Mu X (2006) Beta-catenin is essential for lamination but not neurogenesis in mouse retinal development. Dev Biol 299: 424-437. doi:10.1016/j.ydbio.2006.08.015. PubMed: 16959241. 16959241 PMC3385515

[9] Herranz H, Stamataki E, Feiguin F, Milán M (2006) Self-refinement of Notch activity through the transmembrane protein Crumbs: modulation of gamma-secretase activity. EMBO Rep 7: 297-302. doi:10.1038/sj.embor.7400617. PubMed: 16440003. 16440003 PMC1456882

[10] Mitsuishi Y, Hasegawa H, Matsuo A, Araki W, Suzuki T et al. (2010) Human CRB2 inhibits gamma-secretase cleavage of amyloid precursor protein by binding to the presenilin complex. J Biol Chem 285: 14920-14931. doi:10.1074/jbc.M109.038760. PubMed: 20299451. 20299451 PMC2865292

[11] Ohata S, Aoki R, Kinoshita S, Yamaguchi M, Tsuruoka-Kinoshita S et al. (2011) Dual roles of Notch in regulation of apically restricted mitosis and apicobasal polarity of neuroepithelial cells. Neuron 69: 215-230. doi:10.1016/j.neuron.2010.12.026. PubMed: 21262462. 21262462

[12] Massey-Harroche D, Delgrossi MH, Lane-Guermonprez L, Arsanto JP, Borg JP et al. (2007) Evidence for a molecular link between the tuberous sclerosis complex and the Crumbs complex. Hum Mol Genet 16: 529-536. doi:10.1093/hmg/ddl485. PubMed: 17234746. 17234746

[13] Kim S, Lehtinen MK, Sessa A, Zappaterra MW, Cho SH et al. (2010) The apical complex couples cell fate and cell survival to cerebral cortical development. Neuron 66: 69-84. doi:10.1016/j.neuron.2010.03.019. PubMed: 20399730. 20399730 PMC2872122

[14] Zhao B, Tumaneng K, Guan KL (2011) The Hippo pathway in organ size control, tissue regeneration and stem cell self-renewal. Nat Cell Biol 13: 877-883. doi:10.1038/ncb2303. PubMed: 21808241. 21808241 PMC3987945

[15] Chen CL, Gajewski KM, Hamaratoglu F, Bossuyt W, Sansores-Garcia L et al. (2010) The apical-basal cell polarity determinant Crumbs regulates Hippo signaling in Drosophila. Proc Natl Acad Sci U S A 107: 15810-15815. doi:10.1073/pnas.1004060107. PubMed: 20798049. 20798049 PMC2936591

[16] Robinson BS, Huang J, Hong Y, Moberg KH (2010) Crumbs regulates Salvador/Warts/Hippo signaling in Drosophila via the FERM-domain protein Expanded. Curr Biol 20: 582-590. doi:10.1016/j.cub.2010.03.019. PubMed: 20362445. 20362445 PMC2855393

[17] Varelas X, Samavarchi-Tehrani P, Narimatsu M, Weiss A, Cockburn K et al. (2010) The Crumbs complex couples cell density sensing to Hippo-dependent control of the TGF-beta-SMAD pathway. Dev Cell 19: 831-844. doi:10.1016/j.devcel.2010.11.012. PubMed: 21145499. 21145499

[18] Pellikka M, Tanentzapf G, Pinto M, Smith C, McGlade CJ et al. (2002) Crumbs, the Drosophila homologue of human CRB1/RP12, is essential for photoreceptor morphogenesis. Nature 416: 143-149. doi:10.1038/nature721. PubMed: 11850625. 11850625

[19] Richard M, Roepman R, Aartsen WM, van Rossum AG, den Hollander AI, et al. (2006) Towards understanding CRUMBS function in retinal dystrophies. Hum Mol Genet 15 Spec No 2: R235-243 10.1093/hmg/ddl195 16987889

[20] den Hollander AI, ten Brink JB, de Kok YJ, van Soest S, van den Born LI et al. (1999) Mutations in a human homologue of Drosophila crumbs cause retinitis pigmentosa (RP12). Nat Genet 23: 217-221. doi:10.1038/13848. PubMed: 10508521. 10508521

[21] den Hollander AI, Davis J, van der Velde-Visser SD, Zonneveld MN, Pierrottet CO et al. (2004) CRB1 mutation spectrum in inherited retinal dystrophies. Hum Mutat 24: 355-369. doi:10.1002/humu.20093. PubMed: 15459956. 15459956

[22] Mehalow AK, Kameya S, Smith RS, Hawes NL, Denegre JM et al. (2003) CRB1 is essential for external limiting membrane integrity and photoreceptor morphogenesis in the mammalian retina. Hum Mol Genet 12: 2179-2189. doi:10.1093/hmg/ddg232. PubMed: 12915475. 12915475

[23] Pellissier LP, Alves CH, Quinn PM, Vos RM, Tanimoto N et al. (2013) Targeted Ablation of Crb1 and Crb2 in Retinal Progenitor Cells Mimics Leber Congenital Amaurosis. PLOS Genet (in press). 10.1371/journal.pgen.1003976 PMC3854796 24339791

[24] Zhang SS, Xu X, Liu MG, Zhao H, Soares MB et al. (2006) A biphasic pattern of gene expression during mouse retina development. BMC Dev Biol 6: 48. doi:10.1186/1471-213X-6-48. PubMed: 17044933. 17044933 PMC1633734

[25] Livesey FJ, Furukawa T, Steffen MA, Church GM, Cepko CL (2000) Microarray analysis of the transcriptional network controlled by the photoreceptor homeobox gene Crx. Curr Biol 10: 301-310. doi:10.1016/S0960-9822(00)00379-1. PubMed: 10744971. 10744971

[26] Farjo R, Yu J, Othman MI, Yoshida S, Sheth S et al. (2002) Mouse eye gene microarrays for investigating ocular development and disease. Vision Res 42: 463-470. doi:10.1016/S0042-6989(01)00219-X. PubMed: 11853762. 11853762

[27] Díaz E, Yang YH, Ferreira T, Loh KC, Okazaki Y et al. (2003) Analysis of gene expression in the developing mouse retina. Proc Natl Acad Sci U S A 100: 5491-5496. doi:10.1073/pnas.0831080100. PubMed: 12702772. 12702772 PMC154372

[28] Dorrell MI, Aguilar E, Weber C, Friedlander M (2004) Global gene expression analysis of the developing postnatal mouse retina. Invest Ophthalmol Vis Sci 45: 1009-1019. doi:10.1167/iovs.03-0806. PubMed: 14985324. 14985324

[29] Liu MG, Li H, Xu X, Barnstable CJ, Zhang SS (2008) Comparison of gene expression during in vivo and in vitro postnatal retina development. J Ocul Biol Dis Infor 1: 59-72. doi:10.1007/s12177-008-9009-z. PubMed: 20072636. 20072636 PMC2802513

[30] Gustincich S, Contini M, Gariboldi M, Puopolo M, Kadota K et al. (2004) Gene discovery in genetically labeled single dopaminergic neurons of the retina. Proc Natl Acad Sci U S A 101: 5069-5074. doi:10.1073/pnas.0400913101. PubMed: 15047890. 15047890 PMC387375

[31] Farkas RH, Qian J, Goldberg JL, Quigley HA, Zack DJ (2004) Gene expression profiling of purified rat retinal ganglion cells. Invest Ophthalmol Vis Sci 45: 2503-2513. doi:10.1167/iovs.03-1391. PubMed: 15277470. 15277470

[32] Mu X, Beremand PD, Zhao S, Pershad R, Sun H et al. (2004) Discrete gene sets depend on POU domain transcription factor Brn3b/Brn-3.2/POU4f2 for their expression in the mouse embryonic retina. Development 131: 1197-1210. doi:10.1242/dev.01010. PubMed: 14973295. 14973295

[33] Zhang SS, Xu X, Li J, Liu MG, Zhao H et al. (2005) Comprehensive in silico functional specification of mouse retina transcripts. BMC Genomics 6: 40. doi:10.1186/1471-2164-6-40. PubMed: 15777472. 15777472 PMC1083414

[34] Corbo JC, Cepko CL (2005) A hybrid photoreceptor expressing both rod and cone genes in a mouse model of enhanced S-cone syndrome. PLoS Genet 1: e11. doi:10.1371/journal.pgen.0010011. PubMed: 16110338. 16110338 PMC1186732

[35] Znoiko SL, Rohrer B, Lu K, Lohr HR, Crouch RK et al. (2005) Downregulation of cone-specific gene expression and degeneration of cone photoreceptors in the Rpe65-/- mouse at early ages. Invest Ophthalmol Vis Sci 46: 1473-1479. doi:10.1167/iovs.04-0653. PubMed: 15790918. 15790918

[36] van de Pavert SA, Meuleman J, Malysheva A, Aartsen WM, Versteeg I et al. (2007) A single amino acid substitution (Cys249Trp) in Crb1 causes retinal degeneration and deregulates expression of pituitary tumor transforming gene Pttg1. J Neurosci 27: 564-573. doi:10.1523/JNEUROSCI.3496-06.2007. PubMed: 17234588. 17234588 PMC6672796

[37] Muzumdar MD, Tasic B, Miyamichi K, Li L, Luo L (2007) A global double-fluorescent Cre reporter mouse. Genesis 45: 593-605. doi:10.1002/dvg.20335. PubMed: 17868096. 17868096

[38] Smyth GK (2004) Linear models and empirical bayes methods for assessing differential expression in microarray experiments. Stat Appl Genet Mol Biol 3: Article3 10.2202/1544-6115.1027 16646809

[39] Bossers K, Ylstra B, Brakenhoff RH, Smeets SJ, Verhaagen J et al. (2010) Intensity-based analysis of dual-color gene expression data as an alternative to ratio-based analysis to enhance reproducibility. BMC Genomics 11: 112. doi:10.1186/1471-2164-11-112. PubMed: 20163706. 20163706 PMC2838842

[40] Hochberg Y.BaY (1995) Controlling the False Discovery Rate: A Practical and Powerful Approach to Multiple Testing. J R Stat Soc Series B Stat Methodol 57: 289-300

[41] Edgar R, Domrachev M, Lash AE (2002) Gene Expression Omnibus: NCBI gene expression and hybridization array data repository. Nucleic Acids Res 30: 207-210. doi:10.1093/nar/30.1.207. PubMed: 11752295. 11752295 PMC99122

[42] van de Pavert SA, Sanz AS, Aartsen WM, Vos RM, Versteeg I et al. (2007) Crb1 is a determinant of retinal apical Muller glia cell features. Glia 55: 1486-1497. doi:10.1002/glia.20561. PubMed: 17705196. 17705196

