# Supplementary figures and images for: Microarray and Morphological Analysis of Early Postnatal CRB2 Mutant Retinas on a Pure C57BL/6J Genetic Background
Source: PLoS One. 2013 Dec 6;8(12):e82532. doi: 10.1371/journal.pone.0082532 (PMC3855766; doi:10.1371/journal.pone.0082532)

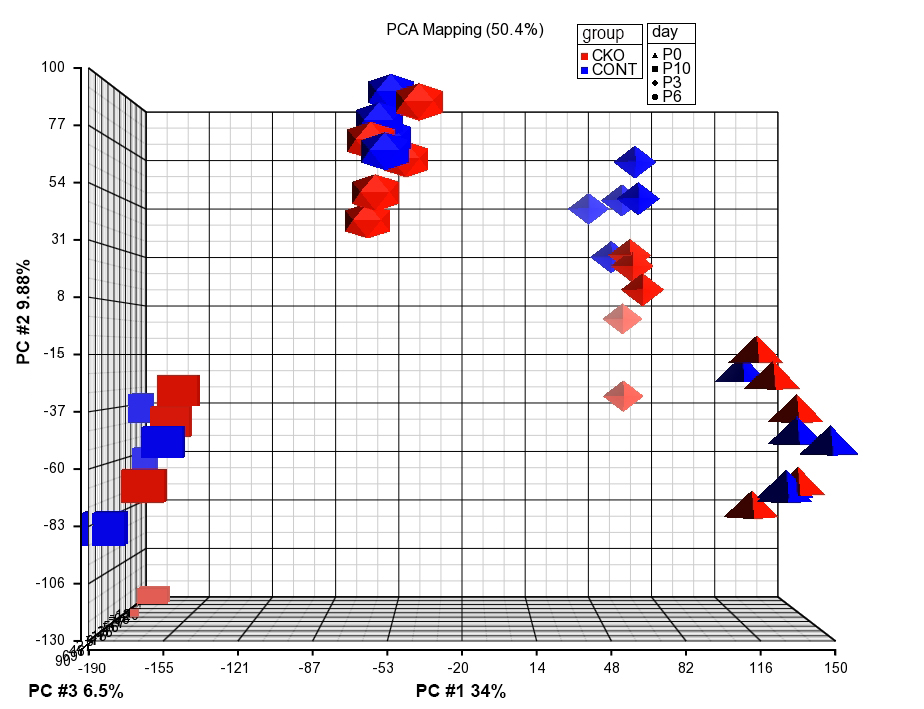

Supplement: Figure S1 — Principal component analysis (PCA) of all probes and all samples. PCA analysis separates samples on the age. Each data point/shape represents a sample, with all the arrays presented on the PCA plot. Crb2 conditional knockout (CKO) (red), and control (CONT) (blue) samples, are distinguished by color, and age P0 (piramide), P32 (tetrahedron), P6 (octahedron), and P10 (cube) are distinguished by shape. Given PC 1 describes the largest amount of data variance (34%), the aggregation of the samples is mostly accounted for the sample age. The percentage values in parentheses indicate the proportion of total variance described by each PC. PC 1 principal component 1 (X-axis); PC 2 principal component 2 (Y-axis); PC 3 principal component 3 (Z-axis). (TIF) [file pone.0082532.s001.tif]

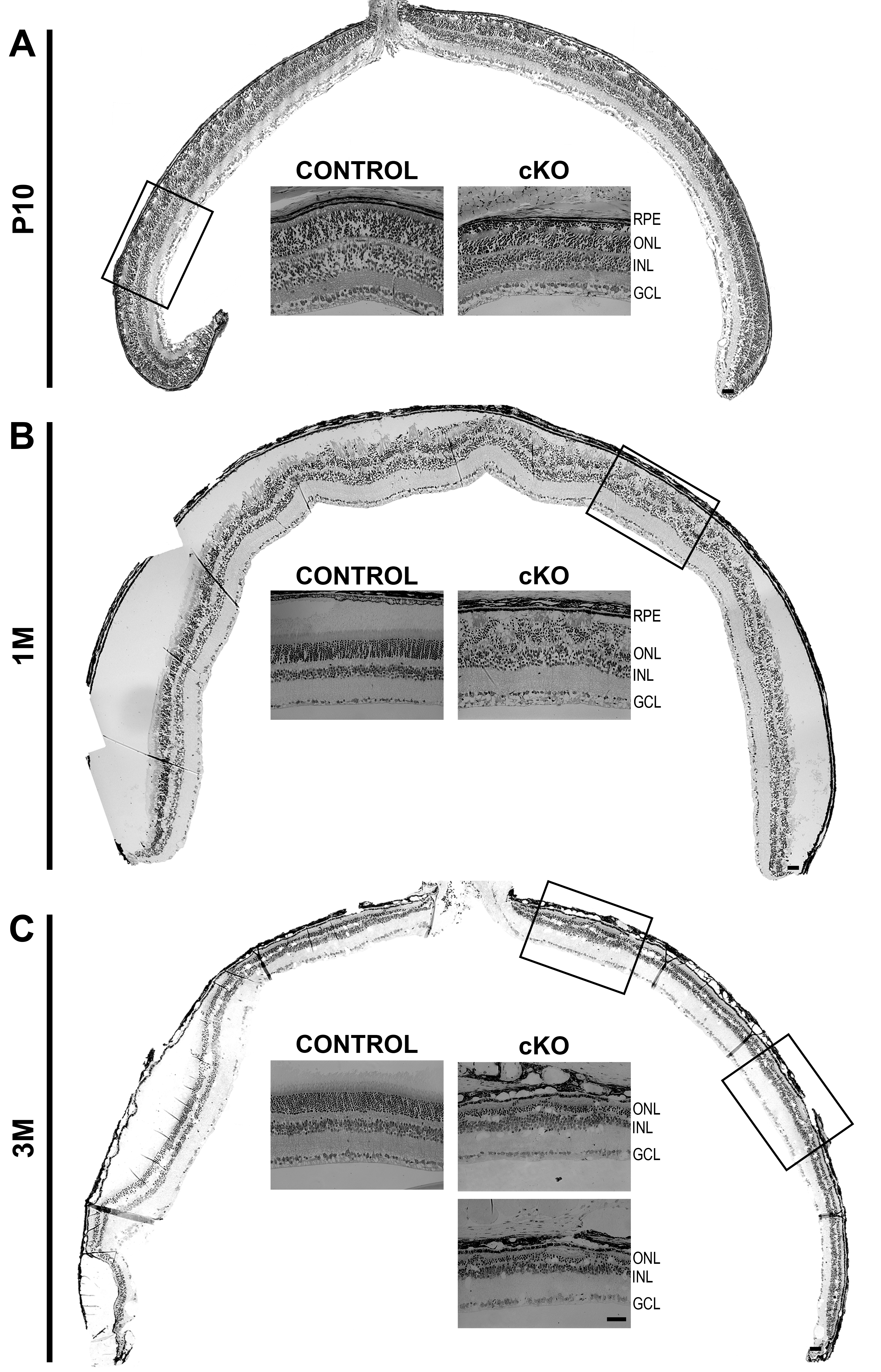

Supplement: Figure S2 — Loss of CRB2 results retinal disorganization in mice C57BL/6J genetic background. Toluidine stained light microscopic pictures of retina sections at different ages, P10 - (A), 1M - (C), 3M - (D). Whole section figures, from the CRB2 null retinas, were mounted from individual pictures using stitching/MosaicJ pluging from ImageJA v1.45b. GCL, ganglion cell layer; INL, inner nuclear layer; ONL, outer nuclear layer. Scale bar: 50 µm. (TIF) [file pone.0082532.s002.tif]
