# Supplementary material for: Microarray and Morphological Analysis of Early Postnatal CRB2 Mutant Retinas on a Pure C57BL/6J Genetic Background
Source: PLoS One. 2013 Dec 6;8(12):e82532. doi: 10.1371/journal.pone.0082532 (PMC3855766; doi:10.1371/journal.pone.0082532)
Supplement: Table S1 — Concentration and integrity of the RNA and cRNA samples used in the microarray (P0 and P3). (DOCX) [file pone.0082532.s003.docx]

**Table S1.**

| Time Point | Group | Sample ID | Abs (nm) | 260nm | 280nm | 260/280 | 230/260 | conc (ng/µl) | µg | RIN | Dye | Cy (pmol/µl) | µg cRNA | pmol Cy per µg cRNA |
| --- | --- | --- | --- | --- | --- | --- | --- | --- | --- | --- | --- | --- | --- | --- |
| P0 | Control | 3 | 2.80 | 6.50 | 3.30 | 1.96 | 2.27 | 260.7 | 7.8 | 9.2 | Cy3 | 2.0 | 6.0 | 10.1 |
| P0 | Control | 7 | 4.20 | 6.10 | 3.10 | 1.98 | 1.47 | 247.3 | 7.4 | 9.5 | Cy3 | 2.8 | 7.3 | 11.4 |
| P0 | Control | 10 | 3.40 | 7.60 | 3.90 | 1.92 | 2.23 | 306.2 | 9.2 | 8.4 | Cy5 | 2.8 | 6.0 | 14.0 |
| P0 | Control | 13 | 2.30 | 5.20 | 2.60 | 1.97 | 2.21 | 209.4 | 6.3 | 9.5 | Cy5 | 3.0 | 6.7 | 13.5 |
| P0 | Control | 24 | 2.30 | 4.30 | 2.20 | 1.94 | 1.85 | 173.6 | 5.2 | 9.1 | Cy5 | 3.0 | 6.0 | 14.9 |
| P0 | cKO | 2 | 2.50 | 5.20 | 2.60 | 1.94 | 2.07 | 209.1 | 6.3 | 9.5 | Cy3 | 3.0 | 7.2 | 12.6 |
| P0 | cKO | 15 | 2.70 | 6.10 | 3.10 | 1.94 | 2.26 | 245.2 | 7.4 | 10.0 | Cy3 | 2.5 | 6.3 | 12.0 |
| P0 | cKO | 20 | 2.50 | 4.98 | 2.50 | 1.96 | 1.94 | 199.5 | 6.0 | 9.7 | Cy3 | 3.1 | 7.3 | 12.7 |
| P0 | cKO | 25 | 4.00 | 4.80 | 2.40 | 1.99 | 1.18 | 193.6 | 5.8 | 8.8 | Cy5 | 2.0 | 5.6 | 10.7 |
| P0 | cKO | 28 | 2.50 | 5.80 | 2.90 | 1.96 | 2.24 | 232.8 | 7.0 | 9.9 | Cy5 | 2.4 | 6.6 | 10.9 |
| P3 | Control | 11 | 2.10 | 4.70 | 2.40 | 1.97 | 2.27 | 190.7 | 5.7 | 10.0 | Cy3 | 3.2 | 6.6 | 14.5 |
| P3 | Control | 21 | 2.90 | 5.50 | 2.70 | 2.05 | 1.92 | 223.6 | 6.7 | 9.5 | Cy3 | 3.3 | 7.4 | 13.3 |
| P3 | Control | 23 | 2.60 | 5.80 | 2.90 | 2.00 | 2.21 | 233.4 | 7.0 | 9.8 | Cy5 | 3.5 | 7.4 | 14.1 |
| P3 | Control | 38 | 2.70 | 5.20 | 2.60 | 2.00 | 1.96 | 211.9 | 6.4 | 9.6 | Cy5 | 3.5 | 7.4 | 14.1 |
| P3 | Control | 43 | 2.40 | 5.30 | 2.60 | 2.03 | 2.19 | 213.8 | 6.4 | 9.3 | Cy5 | 3.7 | 7.5 | 14.8 |
| P3 | cKO | 8 | 1.90 | 3.80 | 1.90 | 1.93 | 1.97 | 153.2 | 4.6 | 8.4 | Cy3 | 2.5 | 6.4 | 11.7 |
| P3 | cKO | 17 | 2.50 | 5.70 | 2.80 | 2.02 | 2.29 | 230.7 | 6.9 | 9.9 | Cy3 | 3.1 | 7.5 | 12.4 |
| P3 | cKO | 22 | 3.20 | 6.80 | 3.40 | 2.00 | 2.18 | 274.7 | 8.2 | 9.9 | Cy3 | 2.9 | 7.1 | 12.2 |
| P3 | cKO | 34 | 2.80 | 6.50 | 3.20 | 1.98 | 2.29 | 260.4 | 7.8 | 9.3 | Cy5 | 4.2 | 8.2 | 15.4 |
| P3 | cKO | 36 | 2.70 | 6.10 | 3.10 | 1.98 | 2.24 | 247.0 | 7.4 | 10.0 | Cy5 | 3.0 | 6.6 | 13.6 |
