# Supplementary material for: Microarray and Morphological Analysis of Early Postnatal CRB2 Mutant Retinas on a Pure C57BL/6J Genetic Background
Source: PLoS One. 2013 Dec 6;8(12):e82532. doi: 10.1371/journal.pone.0082532 (PMC3855766; doi:10.1371/journal.pone.0082532)
Supplement: Table S2 — Concentration and integrity of the RNA and cRNA samples used in the microarray (P6 and P10). (DOCX) [file pone.0082532.s004.docx]

**Table S2.**

| Time Point | Group | Sample ID | Abs (nm) | 260nm | 280nm | 260/280 | 230/260 | conc (ng/µl) | µg | RIN | Dye | Cy (pmol/µl) | µg cRNA | pmol Cy per µg cRNA |
| --- | --- | --- | --- | --- | --- | --- | --- | --- | --- | --- | --- | --- | --- | --- |
| P6 | Control | **11** | 3.10 | 5.00 | 2.40 | 2.03 | 1.59 | 200.5 | 6.0 | 9.4 | Cy3 | 2.5 | 6.3 | 11.9 |
| P6 | Control | **18** | 1.90 | 4.20 | 2.10 | 2.03 | 2.27 | 171.8 | 5.2 | 9.4 | Cy3 | 3.1 | 7.3 | 12.7 |
| P6 | Control | **20** | 2.50 | 4.90 | 2.40 | 2.01 | 1.96 | 199.8 | 6.0 | 9.5 | Cy5 | 3.8 | 7.4 | 15.5 |
| P6 | Control | **22** | 2.30 | 4.70 | 2.30 | 1.97 | 2.12 | 188.9 | 5.7 | 9.2 | Cy5 | 3.7 | 7.0 | 15.9 |
| P6 | Control | **24** | 2.60 | 5.60 | 2.70 | 2.03 | 2.11 | 224.9 | 6.8 | 9.5 | Cy5 | 2.4 | 5.3 | 13.6 |
| P6 | cKO | **1** | 3.20 | 7.10 | 3.60 | 1.97 | 2.22 | 285.8 | 8.6 | 9.5 | Cy3 | 3.6 | 8.1 | 13.3 |
| P6 | cKO | **4** | 2.50 | 5.40 | 2.70 | 1.97 | 2.16 | 218.9 | 6.6 | 9.4 | Cy3 | 3.0 | 7.0 | 12.9 |
| P6 | cKO | **8** | 2.30 | 4.80 | 2.30 | 2.02 | 2.09 | 192.8 | 5.8 | 9.5 | Cy3 | 3.3 | 7.3 | 13.5 |
| P6 | cKO | **13R** | 2.33 | 4.50 | 2.30 | 2.00 | 1.94 | 180.8 | 5.4 | 8.7 | Cy5 | 3.4 | 6.6 | 15.4 |
| P6 | cKO | **27** | 2.10 | 4.20 | 2.10 | 2.01 | 1.93 | 169.2 | 5.1 | 9.6 | Cy5 | 3.8 | 7.1 | 16.1 |
| P10 | Control | **20** | 2.90 | 5.40 | 2.70 | 2.02 | 1.88 | 216.0 | 6.5 | 8.8 | Cy3 | 5.3 | 9.7 | 16.4 |
| P10 | Control | **22** | 3.10 | 7.00 | 3.50 | 2.00 | 2.26 | 281.5 | 8.5 | 9.0 | Cy3 | 3.8 | 8.0 | 14.2 |
| P10 | Control | **32** | 2.60 | 5.90 | 2.90 | 2.00 | 2.23 | 235.3 | 7.1 | 8.4 | Cy5 | 3.5 | 3.7 | 15.6 |
| P10 | Control | **33** | 2.60 | 5.20 | 2.50 | 2.04 | 1.95 | 208.7 | 6.3 | 8.3 | Cy5 | 3.6 | 6.0 | 17.9 |
| P10 | Control | **34** | 2.50 | 5.20 | 2.60 | 2.01 | 2.01 | 208.5 | 6.3 | 9.0 | Cy5 | 4.3 | 7.8 | 16.6 |
| P10 | cKO | **24** | 2.50 | 5.10 | 2.50 | 2.03 | 2.02 | 202.4 | 6.1 | 8.6 | Cy3 | 3.5 | 6.4 | 16.3 |
| P10 | cKO | **25** | 2.60 | 5.60 | 2.80 | 2.03 | 2.13 | 223.9 | 6.7 | 9.2 | Cy3 | 4.4 | 8.3 | 15.9 |
| P10 | cKO | **26** | 1.70 | 3.70 | 1.90 | 1.99 | 2.12 | 148.0 | 4.4 | 9.0 | Cy3 | 3.6 | 7.0 | 15.5 |
| P10 | cKO | **27** | 2.20 | 4.70 | 2.40 | 2.00 | 2.18 | 189.1 | 5.7 | 9.5 | Cy5 | 5.1 | 8.6 | 17.9 |
| P10 | cKO | **29** | 4.90 | 5.50 | 2.70 | 2.01 | 1.12 | 219.2 | 6.6 | 8.5 | Cy5 | 3.8 | 6.5 | 17.6 |
