# Supplementary material for: Microarray and Morphological Analysis of Early Postnatal CRB2 Mutant Retinas on a Pure C57BL/6J Genetic Background
Source: PLoS One. 2013 Dec 6;8(12):e82532. doi: 10.1371/journal.pone.0082532 (PMC3855766; doi:10.1371/journal.pone.0082532)
Supplement: Table S3 — Description of the hybridization pairs and distribution of the samples in the arrays. (DOCX) [file pone.0082532.s005.docx]

**Table S3.**

| Array | Age | Sample ID | Group | Dye | pmol Cy per µg cRNA |
| --- | --- | --- | --- | --- | --- |
| 1 | P0 | 3 | Control | Cy3 | 10.1 |
|  | P0 | 25 | cKO | Cy5 | 10.7 |
| 2 | P0 | 7 | Control | Cy3 | 11.4 |
|  | P0 | 28 | cKO | Cy5 | 10.9 |
| 3 | P0 | 2 | cKO | Cy3 | 12.6 |
|  | P0 | 10 | Control | Cy5 | 14.0 |
| 4 | P0 | 15 | cKO | Cy3 | 12.0 |
|  | P0 | 13 | Control | Cy5 | 13.5 |
| 5 | P0 | 24 | Control | Cy5 | 14.9 |
|  | P0 | 20 | cKO | Cy3 | 12.7 |
| 6 | P3 | 23 | Control | Cy5 | 14.1 |
|  | P3 | 8 | cKO | Cy3 | 11.7 |
| 7 | P3 | 38 | Control | Cy5 | 14.1 |
|  | P3 | 17 | cKO | Cy3 | 12.4 |
| 8 | P3 | 43 | Control | Cy5 | 14.8 |
|  | P3 | 22 | cKO | Cy3 | 12.2 |
| 9 | P3 | 34 | cKO | Cy5 | 15.4 |
|  | P3 | 11 | Control | Cy3 | 14.5 |
| 10 | P3 | 36 | cKO | Cy5 | 13.6 |
|  | P3 | 21 | Control | Cy3 | 13.3 |
| 11 | P6 | 13R | cKO | Cy5 | 15.4 |
|  | P6 | 11 | Control | Cy3 | 11.9 |
| 12 | P6 | 27 | cKO | Cy5 | 16.1 |
|  | P6 | 18 | Control | Cy3 | 12.7 |
| 13 | P6 | 20 | Control | Cy5 | 15.5 |
|  | P6 | 1 | cKO | Cy3 | 13.3 |
| 14 | P6 | 22 | Control | Cy5 | 15.9 |
|  | P6 | 4 | cKO | Cy3 | 12.9 |
| 15 | P6 | 24 | Control | Cy5 | 13.6 |
|  | P6 | 8 | cKO | Cy3 | 13.5 |
| 16 | P10 | 29 | Control | Cy5 | 17.6 |
|  | P10 | 20 | cKO | Cy3 | 16.4 |
| 17 | P10 | 32 | cKO | Cy5 | 16.5 |
|  | P10 | 26 | Control | Cy3 | 15.5 |
| 18 | P10 | 33 | cKO | Cy5 | 17.9 |
|  | P10 | 24 | Control | Cy3 | 16.3 |
| 19 | P10 | 34 | cKO | Cy5 | 16.6 |
|  | P10 | 25 | Control | Cy3 | 15.9 |
| 20 | P10 | 27 | Control | Cy5 | 17.9 |
|  | P10 | 22 | cKO | Cy3 | 14.2 |
