# Supplementary material for: Microarray and Morphological Analysis of Early Postnatal CRB2 Mutant Retinas on a Pure C57BL/6J Genetic Background
Source: PLoS One. 2013 Dec 6;8(12):e82532. doi: 10.1371/journal.pone.0082532 (PMC3855766; doi:10.1371/journal.pone.0082532)
Supplement: Table S4 — qRT-PCR primers used in this study. (DOCX) [file pone.0082532.s006.docx]

**Table S4.**

|  | Gene | Primer ID | Forward | Reverse |
| --- | --- | --- | --- | --- |
| Reference genes | Hprt | 170/171 | CCTAAGATGAGCGCAAGTTGAA | CCACAGGACTAGAACACCTGCTAA |
|  | EF1a | 172/173 | CTGGATGCTCGCCATCAAA | GGCGCTTTTCCTCTTGAAGAA |
|  | RS27a | 174/175 | AAGGTGGATGAAAATGGCAAA | CCATGAAAACTCCCAGCACCA |
| Crumbs Family | mCrb1 | 3/4 | ACAGTGTGTCTGTGCTTAACATCTATAATGT | CGTTCTCCAGAGTAATGTGATTCAAA |
|  | mCrb2 | 534/535 | TCCGGGATCCTACTCCATCTATC | CCAGAAGCACAGTTCCCTTGAG |
|  | mCrb2 | 536/537 | TGCTGGCCTCAGATGTCAGA | CCAGAGAATCCTGCACTGCAA |
|  | mCrb3 | 5/5 | ATTGGGAACCTGAGCGAAGTC | CCCTAAGGCGCCAATTCCTA |
| mTOR | PTEN | 538/539 | TGGATTCGACTTAGACTTGACCT | GCGGTGTCATAATGTCTCTCAG |
|  | PIK3R1 | 542/543 | GCAGAGGGCTACCAGTACAGA | CTGAATCCAAGTGCCACTAAGG |
|  | TSC1 | 546/547 | ACTCTCCCTTCTACCGAGACA | GAGGCTGCCGAATGAGTCTTC |
|  | TSC2 | 550/551 | GAGCTGATTAACTCGGTGGTC | GGCCAGGTCCCTTTCTTCC |
|  | mTOR | 552/553 | ACCGGCACACATTTGAAGAAG | CTCGTTGAGGATCAGCAAGG |
|  | RPS6 | 554/555 | AGCTCCGCACCTTCTATGAGA | GGGAAAACCTTGCTTGTCATTC |
|  | 4BPE1 | 556/557 | GGGGACTACAGCACCACTC | CTCATCGCTGGTAGGGCTA |
|  | eIF4B | 558/559 | ACGGACTTTCTAGCTGAGGAT | CGTCATCATCGTTACTATGCCA |
|  | eIF4E | 560/561 | ACCCCTACCACTAATCCCCC | CAATCGAAGGTTTGCTTGCCA |
| shh | Gli1 | 496/497 | CCAAGCCAACTTTATGTCAGGG | AGCCCGCTTCTTTGTTAATTTGA |
|  | Gli2 | 498/499 | CAACGCCTACTCTCCCAGAC | GAGCCTTGATGTACTGTACCAC |
|  | SMO | 502/503 | GAGCGTAGCTTCCGGGACTA | CTGGGCCGATTCTTGATCTCA |
|  | SHH | 504/505 | AAAGCTGACCCCTTTAGCCTA | TTCGGAGTTTCTTGTGATCTTCC |
|  | PTCH1 | 510/511 | TGTACTGATGCTTGCAGGGT | GAGACACCTCAGGACACGG |
| Notch1 | Notch1 | 386/387 | 5’CCCTTGCTCTGCCTAACGC | GGAGTCCTGGCATCGTTGG |
|  | Hes1 | 388/389 | 5’CCAGCCAGTGTCAACACGA | AATGCCGGGAGCTATCTTTCT |
|  | Hes5 | 390/391 | 5’AGTCCCAAGGAGAAAAACCGA | GCTGTGTTTCAGGTAGCTGAC |
|  | Presenilin1 | 400/401 | 5’GGTGGCTGTTTTATGTCCCAA | CAACCACACCATTGTTGAGGA |
|  | Hey1 | 552/553 | ACGCCCTGGCTATGGACTATC | GGAGGCATCGAGTCCTTCAAT |
|  | Hey2 | 524/525 | CCTGGGCACGCTACAAGCT | GATCCCGACGCCTTTTTTCT |
|  | Heyl | 530/531 | AGATGCAAGCCCGGAAGAA | GACCAATCGTCGCAATTCAGA |
| Wnt | β-catenin | 402/403 | 5’ATGGAGCCGGACAGAAAAGC | 5’CTTGCCACTCAGGGAAGGA |
|  | GSK3β | 434/435 | AAGCGATTTAAGAACCGAGAGC | AGAAATACCGCAGTCGGACTAT |
|  | P120-catenin | 444/445 | GTGGAAACCTACACCGAGGAG | CGTCTAGTGGTCCCATCATCTG |
|  | Kaiso | 446/447 | GAACTCCTTGAATGAACAGCGT | CCCAGCAACTGAGAAGAGC |
|  | Sox2 | 478/479 | AAACCACCAATCCCATCCAAA | CCCCAAAAAGAAGTCCCAAGA |
|  | cMyc | 476/477 | CCACCAGCAGCGACTCTGA | AGCCCGACTCCGACCTCTT |
| Hippo/YAP | YAP | 436/347 | TACTGATGCAGGTACTGCGG | TCAGGGATCTCAAAGGAGGAC |
|  | TAZ | 442/443 | CCGGGTGGGAGATGACCTT | AGGTTCACATGATTCAGAGGCT |
|  | Lats1 | 438/439 | TGGTGACTCTGGGGATAAAGAA | GGGAGTAACTCTGAATCCGAGAC |
|  | Birc5 | 474/475 | CCGAGAACGAGCCTGATTTG | TGCTCCTCTATCGGGTTGTCA |
|  | CTGF | 480/481 | TGTGTGACGAGCCCAAGGA | TTGGGTCTGGGCCAAATGT |
|  | Cyr61 | 464/465 | CTGCGCTAAACAACTCAACGA | GCAGATCCCTTTCAGAGCGG |
