# Supplementary material for: Microarray and Morphological Analysis of Early Postnatal CRB2 Mutant Retinas on a Pure C57BL/6J Genetic Background
Source: PLoS One. 2013 Dec 6;8(12):e82532. doi: 10.1371/journal.pone.0082532 (PMC3855766; doi:10.1371/journal.pone.0082532)
Supplement: Table S5 — Differential gene expression between control and knockout neuroretinas in fold differences, at postnatal day 0. Top 100 genes ranked on their P value given by the students’ t-test (P value) before applying Benjamini–Hochberg (P value bh) method for correct to multiple testing. The expression value to the individual genes for control (CONT) and knockout (CKO) groups (log2 intensity), and the fold differences between control and knockout (FC) are also described in the table. (DOCX) [file pone.0082532.s007.docx]

**Table S5.**

| GeneName | SystematicName | Description | P0 CONT | P0 CKO | FC | P value | P value bh |
| --- | --- | --- | --- | --- | --- | --- | --- |
| LOC100047591 | XM_001478470 | hypothetical protein LOC100047591 (LOC100047591) | 5.06 | 5.34 | 1.22 | 0.00020477 | 0.99998474 |
| Tgfa | NM_031199 | transforming growth factor alpha (Tgfa) | 5.60 | 5.78 | 1.14 | 0.00024542 | 0.99998474 |
| V1re1 | NM_134190 | vomeronasal 1 receptor. E1 (V1re1) | 4.05 | 4.51 | 1.38 | 0.00028090 | 0.99998474 |
| Calml4 | NM_138304 | calmodulin-like 4 (Calml4). transcript variant 1. mRNA [NM_138304] | 5.24 | 5.60 | 1.28 | 0.00089460 | 0.99998474 |
| A_55_P2112510 | A_55_P2112510 | Unknown | 15.24 | 15.39 | 1.11 | 0.00096967 | 0.99998474 |
| NAP060965-1 | NAP060965-1 | Unknown | 7.69 | 7.21 | 0.72 | 0.00115744 | 0.99998474 |
| Capn3 | NM_007601 | calpain 3 (Capn3). transcript variant a | 4.08 | 3.48 | 0.66 | 0.00131212 | 0.99998474 |
| Gm3058 | XM_001475545 | hypothetical protein LOC100040947 (LOC100040947) | 8.85 | 8.40 | 0.73 | 0.00231650 | 0.99998474 |
| Dnlz | NM_026828 | DNL-type zinc finger (Dnlz). transcript variant 1 | 11.13 | 11.32 | 1.14 | 0.00462710 | 0.99998474 |
| 3200002M19Rik | NM_027532 | RIKEN cDNA 3200002M19 gene (3200002M19Rik) | 11.78 | 11.90 | 1.08 | 0.00489026 | 0.99998474 |
| Mlec | NM_175403 | malectin (Mlec) | 7.40 | 7.55 | 1.11 | 0.00496057 | 0.99998474 |
| NAP062079-1 | NAP062079-1 | Kinesin superfamily protein 2C (Fragment) | 5.70 | 5.47 | 0.85 | 0.00559224 | 0.99998474 |
| ENSMUST00000095646 | ENSMUST00000095646 | Putative uncharacterized protein Fragment | 4.09 | 4.37 | 1.21 | 0.00574086 | 0.99998474 |
| 1700024P04Rik | NM_027064 | RIKEN cDNA 1700024P04 gene (1700024P04Rik) | 4.25 | 4.58 | 1.26 | 0.00595546 | 0.99998474 |
| Gpr52 | NM_001146330 | G protein-coupled receptor 52 (Gpr52) | 4.53 | 4.20 | 0.80 | 0.00596235 | 0.99998474 |
| Clybl | NM_029556 | citrate lyase beta like (Clybl) | 6.87 | 7.12 | 1.20 | 0.00611600 | 0.99998474 |
| Morn5 | NM_029309 | MORN repeat containing 5 (Morn5) | 6.81 | 7.00 | 1.15 | 0.00624619 | 0.99998474 |
| Vgf | NM_001039385 | VGF nerve growth factor inducible (Vgf) | 10.61 | 10.41 | 0.87 | 0.00632362 | 0.99998474 |
| Lrrc51 | NM_027053 | leucine rich repeat containing 51 (Lrrc51). transcript variant 1 | 6.39 | 6.60 | 1.15 | 0.00636314 | 0.99998474 |
| Slc41a2 | NM_177388 | solute carrier family 41. member 2 (Slc41a2) | 8.31 | 8.20 | 0.93 | 0.00672973 | 0.99998474 |
| Coq2 | NM_027978 | coenzyme Q2 homolog. prenyltransferase (yeast) (Coq2) | 11.62 | 11.74 | 1.09 | 0.00707903 | 0.99998474 |
| Shisa4 | NM_175259 | shisa homolog 4 (Xenopus laevis) (Shisa4) | 11.45 | 11.56 | 1.08 | 0.00868173 | 0.99998474 |
| Pigk | NM_178016 | phosphatidylinositol glycan anchor biosynthesis. class K (Pigk). transcript variant 2 | 8.53 | 8.74 | 1.16 | 0.00893334 | 0.99998474 |
| 4930562D21Rik | AK016189 | adult male testis cDNA. RIKEN full-length enriched library. clone:4930562D21 | 4.09 | 4.49 | 1.32 | 0.00914549 | 0.99998474 |
| Rps19bp1 | NM_175109 | ribosomal protein S19 binding protein 1 (Rps19bp1) | 12.31 | 12.47 | 1.12 | 0.00934773 | 0.99998474 |
| Dnahc17 | XM_001481301 | dynein. axonemal. heavy chain 17 (Dnahc17) | 3.68 | 4.10 | 1.33 | 0.00971600 | 0.99998474 |
| ENSMUST00000059708 | ENSMUST00000059708 | Testis anion transporter 1 (Anion exchange transporter)(Solute carrier family 26 member 8) | 4.57 | 4.97 | 1.31 | 0.00984376 | 0.99998474 |
| Gsta1 | NM_008181 | glutathione S-transferase. alpha 1 (Ya) (Gsta1) | 5.23 | 5.52 | 1.22 | 0.00993535 | 0.99998474 |
| Hnrnpl | NM_177301 | heterogeneous nuclear ribonucleoprotein L (Hnrnpl) | 10.47 | 10.37 | 0.93 | 0.01004897 | 0.99998474 |
| Scgb1c1 | NM_001099742 | secretoglobin. family 1C. member 1 (Scgb1c1) | 4.89 | 4.55 | 0.79 | 0.01080384 | 0.99998474 |
| Celsr2 | NM_017392 | cadherin. EGF LAG seven-pass G-type receptor 2 (flamingo homolog. Drosophila) (Celsr2). transcript variant 1 | 10.70 | 10.50 | 0.87 | 0.01108310 | 0.99998474 |
| Gm996 | NM_001005424 | predicted gene 996 (Gm996) | 5.45 | 5.12 | 0.80 | 0.01126269 | 0.99998474 |
| Gm3880 | XM_001478363 | hypothetical protein LOC100042516 (LOC100042516) | 5.38 | 5.15 | 0.85 | 0.01126635 | 0.99998474 |
| Kpna7 | NM_001013774 | karyopherin alpha 7 (importin alpha 8) (Kpna7) | 4.89 | 5.12 | 1.18 | 0.01132644 | 0.99998474 |
| LOC675614 | XM_983625 | similar to Chain A. Crystal Structure Of The Mrp14 Complexed With Chaps (LOC675614) | 4.64 | 4.98 | 1.26 | 0.01147793 | 0.99998474 |
| Gzmn | NM_153052 | granzyme N (Gzmn) | 3.52 | 4.13 | 1.53 | 0.01157893 | 0.99998474 |
| 1500009C09Rik | XM_901684 | RIKEN cDNA 1500009C09 gene. transcript variant 2 (1500009C09Rik) | 10.36 | 10.24 | 0.92 | 0.01161230 | 0.99998474 |
| Saps1 | NM_172894 | SAPS domain family. member 1 (Saps1) | 10.02 | 9.91 | 0.92 | 0.01179651 | 0.99998474 |
| Gm5622 | NM_001013816 | predicted gene 5622 (Gm5622) | 4.74 | 4.13 | 0.66 | 0.01306196 | 0.99998474 |
| Ndp | NM_010883 | Norrie disease (pseudoglioma) (human) (Ndp) | 9.62 | 9.49 | 0.91 | 0.01327019 | 0.99998474 |
| Gm5736 | NM_001127686 | predicted gene 5736 (Gm5736) | 3.54 | 3.82 | 1.21 | 0.01343220 | 0.99998474 |
| LOC100045838 | XM_001475393 | hypothetical protein LOC100045838 (LOC100045838) | 4.65 | 4.22 | 0.74 | 0.01385415 | 0.99998474 |
| Parva | NM_020606 | parvin. alpha (Parva) | 9.18 | 9.29 | 1.08 | 0.01410129 | 0.99998474 |
| Sdccag10 | NM_026072 | serologically defined colon cancer antigen 10 (Sdccag10) | 9.97 | 10.08 | 1.08 | 0.01412487 | 0.99998474 |
| Hnf4a | NM_008261 | hepatic nuclear factor 4. alpha (Hnf4a) | 5.12 | 5.46 | 1.27 | 0.01422843 | 0.99998474 |
| Gm3235 | XM_001476682 | hypothetical protein LOC100046595 (LOC100046595) | 4.26 | 4.47 | 1.15 | 0.01422959 | 0.99998474 |
| Ndufs1 | NM_145518 | NADH dehydrogenase (ubiquinone) Fe-S protein 1 (Ndufs1). nuclear gene encoding mitochondrial protein. transcript variant 4 | 13.83 | 13.90 | 1.05 | 0.01428439 | 0.99998474 |
| ENSMUST00000068342 | ENSMUST00000068342 | Putative uncharacterized protein | 11.48 | 11.31 | 0.89 | 0.01448408 | 0.99998474 |
| Plbd2 | NM_023625 | phospholipase B domain containing 2 (Plbd2) | 10.45 | 10.58 | 1.09 | 0.01469086 | 0.99998474 |
| Gsta3 | NM_001077353 | glutathione S-transferase. alpha 3 (Gsta3). transcript variant 1 | 7.37 | 7.75 | 1.30 | 0.01478449 | 0.99998474 |
| Dld | NM_007861 | dihydrolipoamide dehydrogenase (Dld) | 11.84 | 11.95 | 1.07 | 0.01500300 | 0.99998474 |
| St6galnac2 | NM_009180 | ST6 (alpha-N-acetyl-neuraminyl-2.3-beta-galactosyl-1.3)-N-acetylgalactosaminide alpha-2.6-sialyltransferase 2 (St6galnac2) | 9.04 | 9.20 | 1.12 | 0.01519768 | 0.99998474 |
| A_55_P2017597 | A_55_P2017597 | Unknown | 6.45 | 6.14 | 0.81 | 0.01599161 | 0.99998474 |
| Gtsf1l | NM_026630 | gametocyte specific factor 1-like (Gtsf1l) | 5.38 | 5.11 | 0.83 | 0.01614042 | 0.99998474 |
| Tm7sf3 | NM_026281 | transmembrane 7 superfamily member 3 (Tm7sf3) | 6.11 | 6.30 | 1.14 | 0.01629673 | 0.99998474 |
| Hmgn2 | NM_016957 | high mobility group nucleosomal binding domain 2 (Hmgn2) | 16.76 | 16.88 | 1.09 | 0.01633165 | 0.99998474 |
| Sv2c | NM_029210 | synaptic vesicle glycoprotein 2c (Sv2c) | 4.96 | 4.75 | 0.87 | 0.01640575 | 0.99998474 |
| Olfr904 | NM_146801 | olfactory receptor 904 (Olfr904) | 5.32 | 4.96 | 0.78 | 0.01655495 | 0.99998474 |
| Svs3b | NM_173377 | seminal vesicle secretory protein 3B (Svs3b) | 5.13 | 4.87 | 0.83 | 0.01663408 | 0.99998474 |
| Fam179a | NM_177087 | family with sequence similarity 179. member A (Fam179a) | 3.44 | 4.12 | 1.60 | 0.01666850 | 0.99998474 |
| Bbs4 | NM_175325 | Bardet-Biedl syndrome 4 (human) (Bbs4) | 12.22 | 12.36 | 1.10 | 0.01667366 | 0.99998474 |
| 1700112J05Rik | AK018948 | adult male testis cDNA. RIKEN full-length enriched library. clone:1700110A09 | 3.19 | 3.81 | 1.54 | 0.01676865 | 0.99998474 |
| Oas1e | NM_145210 | 2'-5' oligoadenylate synthetase 1E (Oas1e) | 6.16 | 6.39 | 1.17 | 0.01682573 | 0.99998474 |
| BC024814 | NM_146247 | cDNA sequence BC024814 (BC024814) | 10.22 | 10.32 | 1.07 | 0.01747022 | 0.99998474 |
| Ercc8 | NM_028042 | excision repaiross-complementing rodent repair deficiency. complementation group 8 (Ercc8) | 8.12 | 8.36 | 1.17 | 0.01765219 | 0.99998474 |
| Gltpd2 | NM_146020 | glycolipid transfer protein domain containing 2 (Gltpd2) | 5.01 | 4.68 | 0.79 | 0.01802664 | 0.99998474 |
| 4930563J15Rik | AK030044 | adult male testis cDNA. RIKEN full-length enriched library. clone:4932416O05 | 4.17 | 3.81 | 0.78 | 0.01811227 | 0.99998474 |
| D630002G06Rik | NM_172776 | RIKEN cDNA D630002G06 gene (D630002G06Rik) | 3.53 | 3.96 | 1.34 | 0.01819774 | 0.99998474 |
| Lgtn | NM_010709 | ligatin (Lgtn). transcript variant 2 | 10.13 | 10.25 | 1.09 | 0.01821883 | 0.99998474 |
| Wipf3 | NM_001167860 | WAS/WASL interacting protein family. member 3 (Wipf3). transcript variant 1 | 9.54 | 9.41 | 0.91 | 0.01837269 | 0.99998474 |
| Tmem199 | NM_199199 | transmembrane protein 199 (Tmem199) | 10.19 | 10.31 | 1.09 | 0.01886519 | 0.99998474 |
| Irx6 | NM_022428 | Iroquois related homeobox 6 (Drosophila) (Irx6) | 11.95 | 11.80 | 0.90 | 0.01913051 | 0.99998474 |
| Olfr174 | NM_147002 | olfactory receptor 174 (Olfr174) | 4.04 | 3.27 | 0.59 | 0.01957286 | 0.99998474 |
| Zfp664 | NM_001081750 | zinc finger protein 664 (Zfp664) | 14.18 | 14.13 | 0.97 | 0.01963328 | 0.99998474 |
| LOC100047876 | XM_001479198 | hypothetical protein LOC100047876 (LOC100047876) | 4.13 | 4.45 | 1.24 | 0.01977911 | 0.99998474 |
| Rsrc1 | NM_025822 | arginine/serine-rich coiled-coil 1 (Rsrc1) | 9.38 | 9.52 | 1.10 | 0.02011059 | 0.99998474 |
| Pex10 | NM_001042407 | peroxisomal biogenesis factor 10 (Pex10) | 8.10 | 8.21 | 1.08 | 0.02012667 | 0.99998474 |
| AI835438 | AI835438 | UI-M-AQ0-aad-a-02-0-UI.s1 NIH_BMAP_MHI Mus musculus cDNA clone UI-M-AQ0-aad-a-02-0-UI 3' | 5.11 | 5.42 | 1.24 | 0.02016798 | 0.99998474 |
| Hao2 | NM_019545 | hydroxyacid oxidase 2 (Hao2) | 4.83 | 4.52 | 0.80 | 0.02075844 | 0.99998474 |
| Olfr69 | NM_013621 | olfactory receptor 69 (Olfr69) | 5.34 | 5.57 | 1.17 | 0.02086641 | 0.99998474 |
| Gsta3 | NM_001077353 | glutathione S-transferase. alpha 3 (Gsta3). transcript variant 1 | 7.15 | 7.39 | 1.18 | 0.02087704 | 0.99998474 |
| Trip4 | NM_019797 | thyroid hormone receptor interactor 4 (Trip4) | 10.44 | 10.53 | 1.06 | 0.02089154 | 0.99998474 |
| Oxsm | NM_027695 | 3-oxoacyl-ACP synthase. mitochondrial (Oxsm). nuclear gene encoding mitochondrial protein | 8.72 | 8.84 | 1.09 | 0.02113324 | 0.99998474 |
| Krtap3-3 | NM_025524 | keratin associated protein 3-3 (Krtap3-3) | 4.80 | 4.24 | 0.68 | 0.02133220 | 0.99998474 |
| Agxt2l2 | NM_028398 | alanine-glyoxylate aminotransferase 2-like 2 (Agxt2l2) | 7.67 | 7.88 | 1.16 | 0.02153473 | 0.99998474 |
| Rassf6 | NM_028478 | Ras association (RalGDS/AF-6) domain family member 6 (Rassf6) | 4.76 | 5.04 | 1.21 | 0.02158613 | 0.99998474 |
| Olfr811 | NM_146552 | olfactory receptor 811 (Olfr811) | 3.53 | 4.20 | 1.59 | 0.02163746 | 0.99998474 |
| Trub2 | NM_145520 | TruB pseudouridine (psi) synthase homolog 2 (E. coli) (Trub2). transcript variant 1 | 8.56 | 8.99 | 1.35 | 0.02169214 | 0.99998474 |
| ENSMUST00000058450 | ENSMUST00000058450 | Putative uncharacterized protein | 4.39 | 4.69 | 1.23 | 0.02171892 | 0.99998474 |
| Cercam | NM_207298 | cerebral endothelial cell adhesion molecule (Cercam) | 6.86 | 6.45 | 0.75 | 0.02172114 | 0.99998474 |
| Me1 | NM_008615 | malic enzyme 1. NADP(+)-dependent. cytosolic (Me1) | 9.09 | 9.24 | 1.11 | 0.02188046 | 0.99998474 |
| LOC100048053 | XM_001478830 | hypothetical protein LOC100048053 (LOC100048053) | 4.18 | 4.50 | 1.25 | 0.02211119 | 0.99998474 |
| Il1r2 | NM_010555 | interleukin 1 receptor. type II (Il1r2) | 4.29 | 4.71 | 1.34 | 0.02211905 | 0.99998474 |
| 4930443G12Rik | NM_029054 | RIKEN cDNA 4930443G12 gene (4930443G12Rik) | 3.98 | 4.23 | 1.19 | 0.02230815 | 0.99998474 |
| Rnaseh1 | NM_011275 | ribonuclease H1 (Rnaseh1) | 10.30 | 10.40 | 1.07 | 0.02231595 | 0.99998474 |
| Ubb | NM_011664 | ubiquitin B (Ubb) | 17.35 | 17.59 | 1.18 | 0.02247925 | 0.99998474 |
| Olfr678 | NM_146758 | olfactory receptor 678 (Olfr678) | 3.62 | 4.20 | 1.50 | 0.02267083 | 0.99998474 |
| Phldb3 | NM_001102613 | pleckstrin homology-like domain. family B. member 3 (Phldb3) | 6.63 | 6.49 | 0.91 | 0.02279079 | 0.99998474 |
| Sec61b | NM_024171 | Sec61 beta subunit (Sec61b) | 12.13 | 12.24 | 1.08 | 0.02285290 | 0.99998474 |
| Gm13219 | XM_001473580 | hypothetical protein LOC100044956. transcript variant 1 (LOC100044956) | 4.27 | 3.77 | 0.71 | 0.02307938 | 0.99998474 |
