# Supplementary material for: Microarray and Morphological Analysis of Early Postnatal CRB2 Mutant Retinas on a Pure C57BL/6J Genetic Background
Source: PLoS One. 2013 Dec 6;8(12):e82532. doi: 10.1371/journal.pone.0082532 (PMC3855766; doi:10.1371/journal.pone.0082532)
Supplement: Table S6 — Differential gene expression between control and knockout neuroretinas in fold differences, at postnatal day 3. Top 100 genes ranked on their P value given by the students’ t-test (P value) before applying Benjamini–Hochberg (P value bh) method for correct to multiple testing. The expression value to the individual genes for control (CONT) and knockout (CKO) groups (log2 intensity), and the fold differences between control and knockout (FC) are also described in the table. (DOCX) [file pone.0082532.s008.docx]

**Table S6.**

| GeneName | SystematicName | Description | P3 CONT | P3 CKO | FC | P value | P value bh |
| --- | --- | --- | --- | --- | --- | --- | --- |
| NAP060965-1 | NAP060965-1 | Unknown | 7.86 | 7.32 | 0.69 | 1.33E-06 | 0.056788503 |
| Gm3058 | XM_001475545 | hypothetical protein LOC100040947 (LOC100040947) | 9.06 | 8.45 | 0.65 | 0.000130159 | 0.891785973 |
| Ldha | NM_001136069 | lactate dehydrogenase A (Ldha). transcript variant 2 | 17.18 | 17.01 | 0.89 | 0.000274942 | 0.891785973 |
| Flywch2 | NM_029798 | FLYWCH family member 2 (Flywch2) | 11.53 | 11.36 | 0.89 | 0.000298054 | 0.891785973 |
| Cercam | NM_207298 | cerebral endothelial cell adhesion molecule (Cercam) | 6.88 | 6.35 | 0.69 | 0.000394588 | 0.891785973 |
| Wdr72 | NM_001033500 | WD repeat domain 72 (Wdr72) | 5.85 | 5.38 | 0.72 | 0.000400965 | 0.891785973 |
| A_55_P2063471 | A_55_P2063471 | Unknown | 17.21 | 17.00 | 0.86 | 0.000641495 | 0.891785973 |
| Gm3559 | XM_001477150 | hypothetical protein LOC100041875 (LOC100041875) | 7.35 | 7.07 | 0.82 | 0.000719692 | 0.891785973 |
| A_55_P2029791 | A_55_P2029791 | Unknown | 6.39 | 6.21 | 0.88 | 0.000732146 | 0.891785973 |
| St6galnac4 | NM_011373 | ST6 (alpha-N-acetyl-neuraminyl-2.3-beta-galactosyl-1.3)-N-acetylgalactosaminide alpha-2.6-sialyltransferase 4 (St6galnac4) | 8.09 | 7.86 | 0.85 | 0.000741789 | 0.891785973 |
| Gm8566 | XM_989693 | predicted gene. EG667310 (EG667310) | 15.15 | 15.03 | 0.92 | 0.000757403 | 0.891785973 |
| Mrps35 | NM_145573 | mitochondrial ribosomal protein S35 (Mrps35). nuclear gene encoding mitochondrial protein | 11.27 | 11.10 | 0.89 | 0.000909075 | 0.891785973 |
| ENSMUST00000066453 | ENSMUST00000066453 | Putative uncharacterized protein Fragment | 5.29 | 4.96 | 0.79 | 0.000914645 | 0.891785973 |
| 2610028H24Rik | NM_029816 | RIKEN cDNA 2610028H24 gene (2610028H24Rik) | 17.12 | 17.69 | 1.48 | 0.000923483 | 0.891785973 |
| Pde1a | NM_001009978 | phosphodiesterase 1A. calmodulin-dependent (Pde1a). transcript variant 7 | 5.79 | 5.54 | 0.84 | 0.000991859 | 0.891785973 |
| B3gnt4 | NM_198611 | UDP-GlcNAc:betaGal beta-1.3-N-acetylglucosaminyltransferase 4 (B3gnt4) | 6.00 | 5.68 | 0.80 | 0.001043824 | 0.891785973 |
| AI848285 | XM_487043 | similar to hCG1788605 (LOC435145) | 8.89 | 8.73 | 0.89 | 0.001089392 | 0.891785973 |
| Mmp9 | NM_013599 | matrix metallopeptidase 9 (Mmp9) | 5.98 | 5.69 | 0.82 | 0.001241573 | 0.891785973 |
| H2-Q10 | NM_010391 | histocompatibility 2. Q region locus 10 (H2-Q10) | 6.43 | 6.20 | 0.85 | 0.001263372 | 0.891785973 |
| Entpd5 | NM_001026214 | ectonucleoside triphosphate diphosphohydrolase 5 (Entpd5). transcript variant 2 | 9.32 | 9.19 | 0.92 | 0.00127332 | 0.891785973 |
| H2afb2 | XM_888672 | predicted gene. EG624153 (EG624153) | 6.84 | 6.58 | 0.84 | 0.001356892 | 0.891785973 |
| Cited4 | NM_019563 | Cbp/p300-interacting transactivator. with Glu/Asp-rich carboxy-terminal domain. 4 (Cited4) | 7.55 | 7.32 | 0.85 | 0.001506627 | 0.891785973 |
| ENSMUST00000038121 | ENSMUST00000038121 | Putative uncharacterized protein | 7.61 | 7.38 | 0.85 | 0.001507328 | 0.891785973 |
| Mgst2 | NM_174995 | microsomal glutathione S-transferase 2 (Mgst2) | 6.92 | 6.53 | 0.76 | 0.001576677 | 0.891785973 |
| Ankrd39 | NM_026241 | ankyrin repeat domain 39 (Ankrd39) | 10.82 | 10.67 | 0.90 | 0.001578538 | 0.891785973 |
| 5730420D15Rik | AK017586 | 8 days embryo whole body cDNA. RIKEN full-length enriched library. clone:5730420D15 | 4.88 | 4.68 | 0.87 | 0.001585421 | 0.891785973 |
| Pitrm1 | NM_145131 | pitrilysin metallepetidase 1 (Pitrm1) | 12.65 | 12.76 | 1.08 | 0.001589886 | 0.891785973 |
| Romo1 | NM_001164217 | reactive oxygen species modulator 1 (Romo1). nuclear gene encoding mitochondrial protein | 14.52 | 14.33 | 0.88 | 0.001627703 | 0.891785973 |
| Zbtb46 | NM_001162478 | zinc finger and BTB domain containing 46 (Zbtb46). transcript variant 2 | 6.44 | 6.20 | 0.85 | 0.001662049 | 0.891785973 |
| Tesc | NM_021344 | tescalcin (Tesc) | 9.94 | 9.61 | 0.80 | 0.001788673 | 0.891785973 |
| Lrrc1 | NM_172528 | leucine rich repeat containing 1 (Lrrc1). transcript variant 2 | 7.17 | 7.02 | 0.90 | 0.001938844 | 0.891785973 |
| Ccdc115 | NM_027159 | coiled-coil domain containing 115 (Ccdc115) | 12.87 | 12.65 | 0.86 | 0.002074625 | 0.891785973 |
| Tfrc | NM_011638 | transferrin receptor (Tfrc) | 9.82 | 9.50 | 0.80 | 0.002140788 | 0.891785973 |
| Rps10 | NM_025963 | ribosomal protein S10 (Rps10) | 17.54 | 17.27 | 0.83 | 0.002142821 | 0.891785973 |
| Olfr586 | NM_147111 | olfactory receptor 586 (Olfr586) | 5.22 | 5.02 | 0.87 | 0.002180156 | 0.891785973 |
| Gm885 | NM_001033435 | predicted gene 885 (Gm885) | 17.16 | 17.67 | 1.42 | 0.002247837 | 0.891785973 |
| Amz1 | NM_173405 | archaelysin family metallopeptidase 1 (Amz1) | 7.67 | 7.52 | 0.90 | 0.002305178 | 0.891785973 |
| Gm16418 | XM_001475048 | similar to ubiquinol-cytochrome c reductase subunit (LOC100040287) | 14.50 | 14.31 | 0.87 | 0.002329721 | 0.891785973 |
| A_55_P2040559 | A_55_P2040559 | Unknown | 16.94 | 16.78 | 0.89 | 0.00252538 | 0.891785973 |
| Fbxo25 | NM_025785 | F-box protein 25 (Fbxo25) | 4.64 | 4.11 | 0.69 | 0.0025429 | 0.891785973 |
| Scgb1a1 | NM_011681 | secretoglobin. family 1A. member 1 (uteroglobin) (Scgb1a1) | 6.88 | 6.70 | 0.88 | 0.002577483 | 0.891785973 |
| Renbp | NM_023132 | renin binding protein (Renbp). transcript variant 1 | 6.51 | 6.18 | 0.80 | 0.002586076 | 0.891785973 |
| Yars | NM_134151 | tyrosyl-tRNA synthetase (Yars) | 7.47 | 7.29 | 0.88 | 0.00261882 | 0.891785973 |
| Pih1d2 | NM_028300 | PIH1 domain containing 2 (Pih1d2) | 8.18 | 7.99 | 0.88 | 0.002645321 | 0.891785973 |
| Slamf9 | NM_029612 | SLAM family member 9 (Slamf9) | 6.93 | 6.57 | 0.78 | 0.002665466 | 0.891785973 |
| C030037D09Rik | AK034163 | adult male diencephalon cDNA. RIKEN full-length enriched library. clone:9330160M04 | 6.56 | 6.88 | 1.25 | 0.002669374 | 0.891785973 |
| Plekha8 | NM_001164361 | pleckstrin homology domain containing. family A (phosphoinositide binding specific) member 8 (Plekha8). transcript variant 1 | 9.72 | 9.55 | 0.89 | 0.002704293 | 0.891785973 |
| A_55_P2126469 | A_55_P2126469 | Unknown | 16.61 | 16.47 | 0.91 | 0.002732238 | 0.891785973 |
| Gm12260 | XM_905850 | similar to histone H3 (LOC382523) | 15.72 | 15.93 | 1.16 | 0.002756991 | 0.891785973 |
| Tfrc | NM_011638 | transferrin receptor (Tfrc) | 9.79 | 9.37 | 0.75 | 0.002814838 | 0.891785973 |
| COX1 | ENSMUST00000082402 | Cytochrome c oxidase subunit 1 (EC 1.9.3.1)(Cytochrome c oxidase polypeptide I) | 16.88 | 17.62 | 1.67 | 0.002842068 | 0.891785973 |
| LOC100044968 | XM_001473421 | similar to modulator recognition factor 2 (LOC100044968) | 11.78 | 11.95 | 1.13 | 0.002857918 | 0.891785973 |
| LOC100046910 | XM_001477053 | similar to Glycosyltransferase 6 domain containing 1 (LOC100046910) | 4.20 | 4.53 | 1.26 | 0.002872139 | 0.891785973 |
| LOC100046073 | XM_001475530 | hypothetical protein LOC100046073 (LOC100046073) | 8.99 | 9.40 | 1.33 | 0.003173589 | 0.891785973 |
| Cops6 | NM_012002 | COP9 (constitutive photomorphogenic) homolog. subunit 6 (Arabidopsis thaliana) (Cops6) | 13.88 | 13.73 | 0.91 | 0.003199506 | 0.891785973 |
| Lhfp | NM_175386 | lipoma HMGIC fusion partner (Lhfp) | 9.27 | 9.05 | 0.86 | 0.003246309 | 0.891785973 |
| Car8 | NM_007592 | carbonic anhydrase 8 (Car8) | 6.25 | 6.07 | 0.88 | 0.003251049 | 0.891785973 |
| ENSMUST00000075576 | ENSMUST00000075576 | Ankyrin repeat domain-containing protein 33B | 6.21 | 6.58 | 1.29 | 0.00335368 | 0.891785973 |
| Eif3h | NM_080635 | eukaryotic translation initiation factor 3. subunit H (Eif3h) | 16.25 | 16.02 | 0.85 | 0.00336973 | 0.891785973 |
| C030005K06Rik | AK137789 | 16 days neonate thymus cDNA. RIKEN full-length enriched library. clone:A130009K03 | 6.45 | 6.90 | 1.37 | 0.003404441 | 0.891785973 |
| 4933412L11Rik | AK016793 | adult male testis cDNA. RIKEN full-length enriched library. clone:4933412L11 | 5.05 | 5.50 | 1.37 | 0.003438167 | 0.891785973 |
| Nespas | NR_002846 | neuroendocrine secretory protein antisense (Nespas). antisense RNA | 5.50 | 5.73 | 1.18 | 0.00367259 | 0.891785973 |
| Sult4a1 | NM_013873 | sulfotransferase family 4A. member 1 (Sult4a1) | 11.98 | 11.78 | 0.87 | 0.00371482 | 0.891785973 |
| Hist2h3c1 | NM_178216 | histone cluster 2. H3c1 (Hist2h3c1) | 13.81 | 14.07 | 1.20 | 0.003798019 | 0.891785973 |
| Zfp160 | NM_145483 | zinc finger protein 160 (Zfp160) | 9.06 | 9.23 | 1.13 | 0.00386614 | 0.891785973 |
| Rad54b | NM_001039556 | RAD54 homolog B (S. cerevisiae) (Rad54b) | 9.78 | 10.01 | 1.17 | 0.003871451 | 0.891785973 |
| Fundc1 | NM_028058 | FUN14 domain containing 1 (Fundc1) | 12.36 | 12.18 | 0.88 | 0.003884109 | 0.891785973 |
| ENSMUST00000052648 | ENSMUST00000052648 | coiled-coil domain containing 28A | 9.41 | 9.82 | 1.33 | 0.003918921 | 0.891785973 |
| Zfp35 | NM_011755 | zinc finger protein 35 (Zfp35) | 6.91 | 6.67 | 0.85 | 0.004024142 | 0.891785973 |
| Appl1 | NM_145221 | adaptor protein. phosphotyrosine interaction. PH domain and leucine zipper containing 1 (Appl1) | 10.73 | 10.85 | 1.09 | 0.004064316 | 0.891785973 |
| Kcnn2 | NM_080465 | potassium intermediate/small conductance calcium-activated channel. subfamily N. member 2 (Kcnn2) | 8.87 | 9.11 | 1.18 | 0.004124395 | 0.891785973 |
| Gm9454 | XM_001477565 | hypothetical LOC669327 (LOC669327) | 5.68 | 5.41 | 0.83 | 0.004170784 | 0.891785973 |
| Gm15531 | XM_001479917 | similar to metallopanstimulin (LOC100048411) | 16.26 | 16.11 | 0.90 | 0.004222159 | 0.891785973 |
| 4933413G19Rik | NM_027697 | RIKEN cDNA 4933413G19 gene (4933413G19Rik) | 4.50 | 5.05 | 1.46 | 0.004257497 | 0.891785973 |
| Zcchc11 | NM_175472 | zinc finger. CCHC domain containing 11 (Zcchc11) | 10.15 | 10.35 | 1.15 | 0.004322747 | 0.891785973 |
| 4921528O07Rik | NM_030071 | RIKEN cDNA 4921528O07 gene (4921528O07Rik) | 3.42 | 3.97 | 1.47 | 0.004407891 | 0.891785973 |
| Serpina11 | BC024087 | serine (or cysteine) peptidase inhibitor. clade A (alpha-1 antiproteinase. antitrypsin). member 11 | 7.45 | 7.27 | 0.88 | 0.004452225 | 0.891785973 |
| Tymp | NM_138302 | thymidine phosphorylase (Tymp) | 10.20 | 10.02 | 0.88 | 0.004474014 | 0.891785973 |
| Cybrd1 | NM_028593 | cytochrome b reductase 1 (Cybrd1) | 6.50 | 6.27 | 0.85 | 0.004504532 | 0.891785973 |
| LOC100047869 | XM_001479479 | similar to thioredoxin family Trp26 (LOC100047869) | 7.55 | 7.36 | 0.87 | 0.004584489 | 0.891785973 |
| Pex16 | NM_145122 | peroxisomal biogenesis factor 16 (Pex16). transcript variant 1 | 10.59 | 10.40 | 0.88 | 0.004640966 | 0.891785973 |
| Myadml2 | NM_026751 | myeloid-associated differentiation marker-like 2 (Myadml2) | 8.12 | 7.89 | 0.85 | 0.004657357 | 0.891785973 |
| A330050B17Rik | NR_029456 | RIKEN cDNA A330050B17 gene (A330050B17Rik). non-coding RNA | 5.99 | 6.64 | 1.56 | 0.00477359 | 0.891785973 |
| Vsig8 | NM_177723 | V-set and immunoglobulin domain containing 8 (Vsig8). transcript variant 1 | 7.34 | 6.90 | 0.74 | 0.004956158 | 0.891785973 |
| Lass3 | NM_001164201 | LAG1 homolog. ceramide synthase 3 (Lass3) | 7.22 | 6.97 | 0.84 | 0.005011226 | 0.891785973 |
| Sec61g | NM_001109971 | SEC61. gamma subunit (Sec61g). transcript variant 2 | 15.33 | 15.21 | 0.92 | 0.00501572 | 0.891785973 |
| Fkbp14 | NM_153573 | FK506 binding protein 14 (Fkbp14) | 6.61 | 6.40 | 0.86 | 0.005018425 | 0.891785973 |
| ENSMUST00000063754 | ENSMUST00000063754 | Putative uncharacterized protein | 5.91 | 5.63 | 0.83 | 0.005021181 | 0.891785973 |
| Crh | NM_205769 | corticotropin releasing hormone (Crh) | 7.09 | 6.47 | 0.65 | 0.005034862 | 0.891785973 |
| Acsf2 | NM_153807 | acyl-CoA synthetase family member 2 (Acsf2) | 11.23 | 11.73 | 1.41 | 0.00508443 | 0.891785973 |
| Amz1 | NM_173405 | archaelysin family metallopeptidase 1 (Amz1) | 7.65 | 7.46 | 0.88 | 0.005096574 | 0.891785973 |
| Gm4853 | XM_887151 | predicted gene. EG227112 (EG227112) | 15.46 | 15.28 | 0.88 | 0.005130705 | 0.891785973 |
| Spint2 | NM_011464 | serine protease inhibitor. Kunitz type 2 (Spint2). transcript variant 1 | 11.91 | 11.72 | 0.88 | 0.005150139 | 0.891785973 |
| LOC100044525 | XM_001472267 | similar to H3 histone. family 3A (LOC100044525) | 14.80 | 14.59 | 0.87 | 0.005208032 | 0.891785973 |
| LOC100040377 | XM_001474574 | similar to SR protein related family member (rsr-1) (LOC100040377) | 10.77 | 10.59 | 0.88 | 0.005227013 | 0.891785973 |
| Rnu2 | NR_004414 | U2 small nuclear RNA (Rnu2). small nuclear RNA | 9.34 | 8.99 | 0.79 | 0.005228407 | 0.891785973 |
| Ift172 | NM_026298 | intraflagellar transport 172 homolog (Chlamydomonas) (Ift172) | 9.73 | 9.92 | 1.14 | 0.005277038 | 0.891785973 |
| Epn3 | NM_027984 | epsin 3 (Epn3). mRNA [NM_027984] | 11.02 | 11.12 | 1.07 | 0.005313806 | 0.891785973 |
| Dffa | NM_010044 | DNA fragmentation factor. alpha subunit (Dffa). transcript variant 2 | 8.88 | 8.75 | 0.92 | 0.005439404 | 0.891785973 |
| Dapp1 | NM_011932 | dual adaptor for phosphotyrosine and 3-phosphoinositides 1 (Dapp1) | 8.86 | 8.57 | 0.82 | 0.005576524 | 0.891785973 |
