# Supplementary material for: Microarray and Morphological Analysis of Early Postnatal CRB2 Mutant Retinas on a Pure C57BL/6J Genetic Background
Source: PLoS One. 2013 Dec 6;8(12):e82532. doi: 10.1371/journal.pone.0082532 (PMC3855766; doi:10.1371/journal.pone.0082532)
Supplement: Table S7 — Differential gene expression between control and knockout neuroretinas in fold differences, at postnatal day 6. Top 100 genes ranked on their P value given by the students’ t-test (P value) before applying Benjamini–Hochberg (P value bh) method for correct to multiple testing. The expression value to the individual genes for control (CONT) and knockout (CKO) groups (log2 intensity), and the fold differences between control and knockout (FC) are also described in the table. (DOCX) [file pone.0082532.s009.docx]

**Table S7.**

| GeneName | SystematicName | Description | P6 CONT | P6 CKO | FC | P value | P value bh |
| --- | --- | --- | --- | --- | --- | --- | --- |
| Lypd6b | NM_027990 | LY6/PLAUR domain containing 6B (Lypd6b) | 9.39 | 9.04 | 0.78 | 0.000110178 | 0.999991849 |
| Mrps2 | NM_080452 | mitochondrial ribosomal protein S2 (Mrps2). nuclear gene encoding mitochondrial protein. transcript variant 1. mRNA [NM_080452] | 11.45 | 11.60 | 1.11 | 0.000237258 | 0.999991849 |
| Lypd6b | NM_027990 | LY6/PLAUR domain containing 6B (Lypd6b) | 9.50 | 9.17 | 0.80 | 0.000611354 | 0.999991849 |
| ENSMUST00000063585 | ENSMUST00000063585 | Putative uncharacterized protein | 5.18 | 4.84 | 0.79 | 0.000641853 | 0.999991849 |
| 1600014C10Rik | NM_001085385 | RIKEN cDNA 1600014C10 gene (1600014C10Rik). transcript variant 1 | 6.83 | 6.63 | 0.87 | 0.00066672 | 0.999991849 |
| Tceanc | NM_001007577 | transcription elongation factor A (SII) N-terminal and central domain containing (Tceanc) | 5.97 | 5.79 | 0.88 | 0.000825672 | 0.999991849 |
| Tug1 | NR_002321 | taurine upregulated gene 1 (Tug1). transcript variant a. non-coding RNA | 10.42 | 10.57 | 1.11 | 0.000829414 | 0.999991849 |
| Usmg5 | NM_023211 | upregulated during skeletal muscle growth 5 (Usmg5) | 15.50 | 15.41 | 0.95 | 0.000925509 | 0.999991849 |
| Rpl23 | NM_022891 | ribosomal protein L23 (Rpl23) | 16.31 | 16.45 | 1.10 | 0.000928098 | 0.999991849 |
| Rasl12 | NM_001033158 | RAS-like. family 12 (Rasl12). transcript variant 1 | 8.95 | 8.82 | 0.91 | 0.001045609 | 0.999991849 |
| Ccdc113 | NM_172914 | coiled-coil domain containing 113 (Ccdc113) | 11.03 | 10.89 | 0.91 | 0.001390406 | 0.999991849 |
| Sgcb | NM_011890 | sarcoglycan. beta (dystrophin-associated glycoprotein) (Sgcb) | 9.43 | 9.27 | 0.90 | 0.001507023 | 0.999991849 |
| ENSMUST00000075859 | ENSMUST00000075859 | Olfactory receptor Olfr1354 Fragment | 5.03 | 4.84 | 0.87 | 0.001586279 | 0.999991849 |
| Tspan12 | NM_173007 | tetraspanin 12 (Tspan12) | 10.11 | 9.93 | 0.89 | 0.001605633 | 0.999991849 |
| Hhex | NM_008245 | hematopoietically expressed homeobox (Hhex) | 6.97 | 6.68 | 0.82 | 0.001930318 | 0.999991849 |
| Ccdc106 | NM_146178 | coiled-coil domain containing 106 (Ccdc106) | 11.50 | 11.36 | 0.90 | 0.001933456 | 0.999991849 |
| Gm3058 | XM_001475545 | hypothetical protein LOC100040947 (LOC100040947) | 8.55 | 8.05 | 0.71 | 0.002411137 | 0.999991849 |
| Hoxd3 | NM_010468 | homeobox D3 (Hoxd3) | 4.49 | 4.22 | 0.83 | 0.002608346 | 0.999991849 |
| Zeb2 | NM_015753 | zinc finger E-box binding homeobox 2 (Zeb2). transcript variant 2 | 8.56 | 8.76 | 1.14 | 0.002736136 | 0.999991849 |
| 2810422O20Rik | NM_027279 | RIKEN cDNA 2810422O20 gene (2810422O20Rik) | 9.67 | 9.51 | 0.90 | 0.002736382 | 0.999991849 |
| Myc | NM_010849 | myelocytomatosis oncogene (Myc) | 8.33 | 8.50 | 1.12 | 0.002882713 | 0.999991849 |
| Degs2 | NM_027299 | degenerative spermatocyte homolog 2 (Drosophila). lipid desaturase (Degs2). transcript variant 1 | 4.79 | 4.44 | 0.78 | 0.003004947 | 0.999991849 |
| Pfkfb3 | NM_133232 | 6-phosphofructo-2-kinase/fructose-2.6-biphosphatase 3 (Pfkfb3) | 5.52 | 4.98 | 0.69 | 0.00308474 | 0.999991849 |
| Slco2b1 | NM_175316 | solute carrier organic anion transporter family. member 2b1 (Slco2b1) | 8.07 | 7.79 | 0.82 | 0.003186231 | 0.999991849 |
| Pou6f1 | NM_010127 | POU domain. class 6. transcription factor 1 (Pou6f1) | 10.90 | 11.03 | 1.09 | 0.003234517 | 0.999991849 |
| ENSMUST00000109565 | ENSMUST00000109565 | G-protein coupled receptor 98 Precursor (Monogenic audiogenic seizure susceptibility protein 1)(Very large G-protein coupled receptor 1)(Neurepin) | 4.57 | 4.81 | 1.18 | 0.003262297 | 0.999991849 |
| Lypd6b | NM_027990 | LY6/PLAUR domain containing 6B (Lypd6b) | 9.08 | 8.73 | 0.79 | 0.003444454 | 0.999991849 |
| Camk2n1 | NM_025451 | calcium/calmodulin-dependent protein kinase II inhibitor 1 (Camk2n1) | 12.08 | 11.94 | 0.91 | 0.003706402 | 0.999991849 |
| Lypd6b | NM_027990 | LY6/PLAUR domain containing 6B (Lypd6b) | 9.34 | 9.05 | 0.82 | 0.003709985 | 0.999991849 |
| Tbc1d10c | NM_178650 | TBC1 domain family. member 10c (Tbc1d10c) | 7.88 | 8.01 | 1.09 | 0.00375217 | 0.999991849 |
| Sfrs11 | NM_026989 | splicing factor. arginine/serine-rich 11 (Sfrs11). transcript variant 3 | 13.09 | 12.82 | 0.83 | 0.00377532 | 0.999991849 |
| Rspo2 | NM_172815 | R-spondin 2 homolog (Xenopus laevis) (Rspo2) | 8.97 | 9.18 | 1.16 | 0.003880065 | 0.999991849 |
| Rasl12 | NM_001033158 | RAS-like. family 12 (Rasl12). transcript variant 1 | 8.83 | 8.63 | 0.87 | 0.003916974 | 0.999991849 |
| 1500031L02Rik | NM_025892 | RIKEN cDNA 1500031L02 gene (1500031L02Rik) | 13.08 | 13.01 | 0.96 | 0.003967067 | 0.999991849 |
| ENSMUST00000060360 | ENSMUST00000060360 | Uncharacterized protein C17orf47 homolog | 4.37 | 4.66 | 1.22 | 0.004225806 | 0.999991849 |
| Rfx5 | NM_017395 | regulatory factor X. 5 (influences HLA class II expression) (Rfx5) | 5.37 | 5.61 | 1.18 | 0.004302256 | 0.999991849 |
| Igf2bp3 | NM_023670 | insulin-like growth factor 2 mRNA binding protein 3 (Igf2bp3) | 8.48 | 8.63 | 1.11 | 0.004477997 | 0.999991849 |
| Ankrd33 | NM_144790 | ankyrin repeat domain 33 (Ankrd33) | 13.30 | 13.16 | 0.91 | 0.004791839 | 0.999991849 |
| Kbtbd11 | NM_029116 | kelch repeat and BTB (POZ) domain containing 11 (Kbtbd11) | 11.75 | 11.49 | 0.83 | 0.00516125 | 0.999991849 |
| Tpm3 | NM_022314 | tropomyosin 3. gamma (Tpm3) | 8.51 | 8.25 | 0.84 | 0.005230578 | 0.999991849 |
| Fxc1 | NM_019502 | fractured callus expressed transcript 1 (Fxc1) | 13.62 | 13.43 | 0.88 | 0.005308394 | 0.999991849 |
| LOC100046616 | XM_001476512 | similar to aquaporin 5 (LOC100046616) | 7.02 | 6.86 | 0.90 | 0.005312383 | 0.999991849 |
| Lypd6b | NM_027990 | LY6/PLAUR domain containing 6B (Lypd6b) | 9.20 | 8.91 | 0.82 | 0.005396056 | 0.999991849 |
| rp9 | NM_018739 | retinitis pigmentosa 9 (human) (rp9) | 12.36 | 12.16 | 0.87 | 0.005489525 | 0.999991849 |
| Frg1 | NM_013522 | FSHD region gene 1 (Frg1) | 11.26 | 10.98 | 0.82 | 0.005497687 | 0.999991849 |
| 5330426P16Rik | NR_028300 | RIKEN cDNA 5330426P16 gene (5330426P16Rik). non-coding RNA | 7.65 | 7.50 | 0.90 | 0.005639445 | 0.999991849 |
| A_55_P2175381 | A_55_P2175381 | Unknown | 16.44 | 16.33 | 0.93 | 0.005692898 | 0.999991849 |
| Cdc27 | NM_145436 | cell division cycle 27 homolog (S. cerevisiae) (Cdc27) | 9.33 | 9.21 | 0.92 | 0.005808945 | 0.999991849 |
| Lypd6b | NM_027990 | LY6/PLAUR domain containing 6B (Lypd6b) | 9.40 | 9.07 | 0.79 | 0.005853341 | 0.999991849 |
| E230012P03 | AK054025 | 2 days pregnant adult female oviduct cDNA. RIKEN full-length enriched library. clone:E230012P03 | 6.19 | 6.46 | 1.21 | 0.006295273 | 0.999991849 |
| LOC638088 | XM_913981 | similar to Igh protein (LOC638088) | 4.15 | 3.66 | 0.71 | 0.006355586 | 0.999991849 |
| ENSMUST00000108794 | ENSMUST00000108794 | Obscurin (EC 2.7.11.1)(Obscurin-myosin light chain kinase)(Obscurin-MLCK)(Obscurin-RhoGEF) | 5.00 | 5.21 | 1.16 | 0.006361777 | 0.999991849 |
| Tex13a | NM_026469 | testis expressed 13A (Tex13a) | 3.70 | 4.04 | 1.27 | 0.006914113 | 0.999991849 |
| Mmab | NM_029956 | methylmalonic aciduria (cobalamin deficiency) type B homolog (human) (Mmab). nuclear gene encoding mitochondrial protein | 7.64 | 7.51 | 0.92 | 0.007115726 | 0.999991849 |
| Klhl22 | XM_001475578 | hypothetical protein LOC100046098 (LOC100046098) | 5.03 | 5.24 | 1.16 | 0.007238225 | 0.999991849 |
| Ppp2r3d | NM_001163415 | protein phosphatase 2 (formerly 2A). regulatory subunit B''. delta (Ppp2r3d). transcript variant 1 | 8.53 | 8.69 | 1.11 | 0.007431172 | 0.999991849 |
| Etfb | NM_026695 | electron transferring flavoprotein. beta polypeptide (Etfb) | 13.64 | 13.53 | 0.93 | 0.007495696 | 0.999991849 |
| Gm1110 | XM_356144 | gene model 1110. (NCBI) (Gm1110) | 4.29 | 4.73 | 1.35 | 0.007560772 | 0.999991849 |
| Lypd6b | NM_027990 | LY6/PLAUR domain containing 6B (Lypd6b) | 9.19 | 8.88 | 0.81 | 0.007959325 | 0.999991849 |
| Gm6720 | XM_891536 | predicted gene. EG626920 (EG626920) | 12.21 | 12.09 | 0.92 | 0.008069307 | 0.999991849 |
| Btnl1 | NM_001111094 | butyrophilin-like 1 (Btnl1) | 5.35 | 5.00 | 0.79 | 0.008084822 | 0.999991849 |
| Gng12 | NM_025278 | guanine nucleotide binding protein (G protein). gamma 12 (Gng12) | 8.58 | 8.44 | 0.91 | 0.008241583 | 0.999991849 |
| Olfr412 | NM_001011851 | olfactory receptor 412 (Olfr412) | 4.68 | 5.02 | 1.27 | 0.008383578 | 0.999991849 |
| Mrpl14 | NM_026732 | mitochondrial ribosomal protein L14 (Mrpl14). nuclear gene encoding mitochondrial protein | 14.45 | 14.35 | 0.93 | 0.008419596 | 0.999991849 |
| Srd5a2 | NM_053188 | steroid 5 alpha-reductase 2 (Srd5a2) | 5.15 | 5.36 | 1.16 | 0.008551024 | 0.999991849 |
| Glt8d1 | NM_029626 | glycosyltransferase 8 domain containing 1 (Glt8d1). transcript variant 1 | 12.59 | 12.69 | 1.07 | 0.008841092 | 0.999991849 |
| AU015791 | AK139027 | adult male aorta and vein cDNA. RIKEN full-length enriched library. clone:A530083O18 | 5.94 | 5.64 | 0.81 | 0.008916033 | 0.999991849 |
| ENSMUST00000098889 | ENSMUST00000098889 | Unknown | 5.01 | 4.54 | 0.72 | 0.009633549 | 0.999991849 |
| Slc5a9 | NM_145551 | solute carrier family 5 (sodium/glucose cotransporter). member 9 (Slc5a9) | 3.83 | 3.58 | 0.84 | 0.009999806 | 0.999991849 |
| Traf2 | NM_009422 | TNF receptor-associated factor 2 (Traf2) | 12.65 | 12.56 | 0.94 | 0.010078739 | 0.999991849 |
| ENSMUST00000099432 | ENSMUST00000099432 | uncharacterized protein | 8.96 | 9.15 | 1.14 | 0.01035273 | 0.999991849 |
| Olfr290 | NM_146416 | olfactory receptor 290 (Olfr290) | 4.06 | 3.49 | 0.67 | 0.010430321 | 0.999991849 |
| Gm606 | NM_001013761 | predicted gene 606 (Gm606) | 4.70 | 4.48 | 0.86 | 0.010552906 | 0.999991849 |
| ENSMUST00000113891 | ENSMUST00000113891 | Eukaryotic peptide chain release factor GTP-binding subunit ERF3B (Eukaryotic peptide chain release factor subunit 3b)(eRF3b)(G1 to S phase transition protein 2 homolog) | 7.07 | 7.27 | 1.15 | 0.010650034 | 0.999991849 |
| Gm16514 | XM_001472599 | similar to ribosomal protein L35a (LOC100039303) | 15.23 | 15.11 | 0.92 | 0.011102077 | 0.999991849 |
| Hnrnph2 | NM_019868 | heterogeneous nuclear ribonucleoprotein H2 (Hnrnph2) | 11.98 | 12.13 | 1.11 | 0.011184589 | 0.999991849 |
| Esyt1 | NM_011843 | extended synaptotagmin-like protein 1 (Esyt1) | 8.53 | 8.42 | 0.93 | 0.011277119 | 0.999991849 |
| Rps6kb2 | NM_021485 | ribosomal protein S6 kinase. polypeptide 2 (Rps6kb2) | 9.35 | 9.52 | 1.12 | 0.011597287 | 0.999991849 |
| Gm5468 | NR_027376 | predicted gene 5468 (Gm5468). non-coding RNA [NR_027376] | 7.61 | 7.40 | 0.87 | 0.011709492 | 0.999991849 |
| A1cf | NM_001081074 | APOBEC1 complementation factor (A1cf) | 4.49 | 4.15 | 0.79 | 0.011750825 | 0.999991849 |
| LOC100042220 | XM_001477167 | hypothetical protein LOC100042220 (LOC100042220) | 12.50 | 12.91 | 1.33 | 0.011826736 | 0.999991849 |
| Lyl1 | NM_008535 | lymphoblastomic leukemia 1 (Lyl1) | 7.93 | 7.58 | 0.78 | 0.011836392 | 0.999991849 |
| LOC16697 | NR_026831 | keratin associated protein LOC16697 (LOC16697). non-coding RNA | 7.52 | 7.68 | 1.12 | 0.011944149 | 0.999991849 |
| Lypd6b | NM_027990 | LY6/PLAUR domain containing 6B (Lypd6b) | 9.09 | 8.76 | 0.79 | 0.01197508 | 0.999991849 |
| Rasl12 | NM_001033158 | RAS-like. family 12 (Rasl12). transcript variant 1 | 8.80 | 8.65 | 0.90 | 0.012033847 | 0.999991849 |
| Galnt10 | NM_134189 | UDP-N-acetyl-alpha-D-galactosamine:polypeptide N-acetylgalactosaminyltransferase 10 (Galnt10) | 12.21 | 12.07 | 0.91 | 0.0120476 | 0.999991849 |
| Aldh3a1 | NM_007436 | aldehyde dehydrogenase family 3. subfamily A1 (Aldh3a1). transcript variant 1 | 6.18 | 6.03 | 0.90 | 0.012064938 | 0.999991849 |
| F630110N24Rik | NM_028657 | RIKEN cDNA F630110N24 gene (F630110N24Rik) | 6.97 | 6.79 | 0.88 | 0.012277457 | 0.999991849 |
| 0610007P14Rik | NM_021446 | RIKEN cDNA 0610007P14 gene (0610007P14Rik) | 13.69 | 13.61 | 0.94 | 0.012715146 | 0.999991849 |
| Gm4121 | XM_001472111 | hypothetical protein LOC100044458 (LOC100044458) | 9.32 | 9.50 | 1.14 | 0.012781487 | 0.999991849 |
| Gm11589 | XM_001477539 | similar to Rpl17 protein. transcript variant 1 (LOC100048838) | 15.62 | 15.53 | 0.94 | 0.013029322 | 0.999991849 |
| Rasl12 | NM_001033158 | RAS-like. family 12 (Rasl12). transcript variant 1 | 8.86 | 8.68 | 0.88 | 0.013043328 | 0.999991849 |
| Il13ra2 | NM_008356 | interleukin 13 receptor. alpha 2 (Il13ra2) | 6.49 | 6.63 | 1.10 | 0.013183366 | 0.999991849 |
| Mpo | NM_010824 | myeloperoxidase (Mpo). nuclear gene encoding mitochondrial protein | 4.81 | 5.12 | 1.25 | 0.013312163 | 0.999991849 |
| Spag1 | NM_012031 | sperm associated antigen 1 (Spag1) | 7.26 | 7.44 | 1.14 | 0.013388524 | 0.999991849 |
| A_55_P2053541 | A_55_P2053541 | Unknown | 15.82 | 15.77 | 0.96 | 0.013458359 | 0.999991849 |
| Ikzf1 | NM_001025597 | IKAROS family zinc finger 1 (Ikzf1). transcript variant 1 | 4.69 | 5.05 | 1.28 | 0.013633695 | 0.999991849 |
| Gm15246 | XM_001475551 | similar to Ser/Arg-related nuclear matrix protein (LOC100040950) | 3.82 | 4.34 | 1.44 | 0.013660838 | 0.999991849 |
| Tug1 | NR_002321 | taurine upregulated gene 1 (Tug1). transcript variant a. non-coding RNA | 10.32 | 10.49 | 1.13 | 0.014065452 | 0.999991849 |
| Gm347 | NM_001005420 | predicted gene 347 (Gm347) | 10.58 | 10.73 | 1.11 | 0.014097895 | 0.999991849 |
