# Supplementary material for: Microarray and Morphological Analysis of Early Postnatal CRB2 Mutant Retinas on a Pure C57BL/6J Genetic Background
Source: PLoS One. 2013 Dec 6;8(12):e82532. doi: 10.1371/journal.pone.0082532 (PMC3855766; doi:10.1371/journal.pone.0082532)
Supplement: Table S8 — Differential gene expression between control and knockout neuroretinas in fold differences, at postnatal day 10. Top 100 genes ranked on their P value given by the students’ t-test (P value) before applying Benjamini–Hochberg (P value bh) method for correct to multiple testing. The expression value to the individual genes for control (CONT) and knockout (CKO) groups (log2 intensity), and the fold differences between control and knockout (FC) are also described in the table. (DOCX) [file pone.0082532.s010.docx]

**Table S8.**

| GeneName | SystematicName | Description | P10 CONT | P10 CKO | FC | P value | P value bh |
| --- | --- | --- | --- | --- | --- | --- | --- |
| Gm5811 | XM_001474656 | similar to LOC635138 protein (LOC545175) | 10.88 | 11.03 | 1.11 | 8.58E-05 | 0.999976933 |
| Ndufb11 | NM_019435 | NADH dehydrogenase (ubiquinone) 1 beta subcomplex. 11 (Ndufb11) | 8.42 | 8.23 | 0.88 | 0.000209282 | 0.999976933 |
| Orai1 | NM_175423 | ORAI calcium release-activated calcium modulator 1 (Orai1) | 8.00 | 7.79 | 0.86 | 0.000212838 | 0.999976933 |
| Hrasls | NM_013751 | HRAS-like suppressor (Hrasls) | 8.67 | 8.40 | 0.83 | 0.000414922 | 0.999976933 |
| Mki67 | NM_001081117 | antigen identified by monoclonal antibody Ki 67 (Mki67) | 7.90 | 8.45 | 1.46 | 0.000466712 | 0.999976933 |
| P2rx5 | NM_033321 | purinergic receptor P2X. ligand-gated ion channel. 5 (P2rx5) | 7.43 | 7.11 | 0.80 | 0.000470488 | 0.999976933 |
| Zfp503 | NM_145459 | zinc finger protein 503 (Zfp503) | 9.70 | 9.34 | 0.78 | 0.000506882 | 0.999976933 |
| Pcolce2 | NM_029620 | procollagen C-endopeptidase enhancer 2 (Pcolce2) | 7.04 | 6.57 | 0.72 | 0.000559795 | 0.999976933 |
| Cav2 | NM_016900 | caveolin 2 (Cav2) | 9.33 | 8.92 | 0.75 | 0.000610581 | 0.999976933 |
| Ehd4 | NM_133838 | EH-domain containing 4 (Ehd4) | 12.35 | 12.09 | 0.83 | 0.00064004 | 0.999976933 |
| Inpp4b | NM_001024617 | inositol polyphosphate-4-phosphatase. type II (Inpp4b) | 9.36 | 9.17 | 0.87 | 0.000750699 | 0.999976933 |
| Aatf | NM_019816 | apoptosis antagonizing transcription factor (Aatf) | 9.42 | 9.36 | 0.96 | 0.000778397 | 0.999976933 |
| Zcchc17 | NM_153160 | zinc finger. CCHC domain containing 17 (Zcchc17) | 14.05 | 13.98 | 0.96 | 0.000849813 | 0.999976933 |
| St3gal1 | NM_009177 | ST3 beta-galactoside alpha-2.3-sialyltransferase 1 (St3gal1) | 9.10 | 8.73 | 0.77 | 0.000860208 | 0.999976933 |
| Rbm3 | NM_016809 | RNA binding motif protein 3 (Rbm3). transcript variant 1 | 5.57 | 6.02 | 1.37 | 0.000921201 | 0.999976933 |
| Slc4a3 | NM_009208 | solute carrier family 4 (anion exchanger). member 3 (Slc4a3) | 13.46 | 13.21 | 0.84 | 0.000997003 | 0.999976933 |
| Zfp503 | NM_145459 | zinc finger protein 503 (Zfp503) | 9.82 | 9.49 | 0.80 | 0.001141859 | 0.999976933 |
| Cdk14 | NM_011074 | cyclin-dependent kinase 14 (Cdk14) | 13.54 | 13.38 | 0.89 | 0.001224505 | 0.999976933 |
| Magt1 | NM_025952 | magnesium transporter 1 (Magt1) | 8.04 | 8.36 | 1.25 | 0.001306514 | 0.999976933 |
| Entpd3 | NM_178676 | ectonucleoside triphosphate diphosphohydrolase 3 (Entpd3) | 7.92 | 7.61 | 0.81 | 0.001319352 | 0.999976933 |
| Cav2 | NM_016900 | caveolin 2 (Cav2) | 9.27 | 8.81 | 0.73 | 0.00142905 | 0.999976933 |
| Adcy5 | NM_001012765 | adenylate cyclase 5 (Adcy5) | 10.14 | 9.98 | 0.90 | 0.001431536 | 0.999976933 |
| Cav2 | NM_016900 | caveolin 2 (Cav2) | 9.44 | 9.01 | 0.74 | 0.001490084 | 0.999976933 |
| Rmi1 | NM_001168248 | RMI1. RecQ mediated genome instability 1. homolog (S. cerevisiae) (Rmi1). transcript variant 1 | 8.37 | 8.20 | 0.89 | 0.001502686 | 0.999976933 |
| Tsc22d3 | NM_001077364 | TSC22 domain family. member 3 (Tsc22d3) | 12.17 | 12.01 | 0.89 | 0.001531026 | 0.999976933 |
| Lgsn | NM_153601 | lengsin. lens protein with glutamine synthetase domain (Lgsn) | 8.18 | 7.91 | 0.83 | 0.001596484 | 0.999976933 |
| Insrr | NM_011832 | insulin receptor-related receptor (Insrr) | 8.45 | 8.21 | 0.85 | 0.001748212 | 0.999976933 |
| Zfp503 | NM_145459 | zinc finger protein 503 (Zfp503) | 9.53 | 9.22 | 0.81 | 0.001763769 | 0.999976933 |
| Clu | NM_013492 | clusterin (Clu) | 15.25 | 14.78 | 0.72 | 0.001808008 | 0.999976933 |
| Ptprr | NM_011217 | protein tyrosine phosphatase. receptor type. R (Ptprr). transcript variant 1 | 10.25 | 10.03 | 0.86 | 0.001956044 | 0.999976933 |
| Zfp503 | NM_145459 | zinc finger protein 503 (Zfp503) | 9.66 | 9.32 | 0.79 | 0.002209382 | 0.999976933 |
| A_55_P2082057 | A_55_P2082057 | Unknown | 16.62 | 16.44 | 0.88 | 0.002213506 | 0.999976933 |
| Wdr5b | NM_027113 | WD repeat domain 5B (Wdr5b) | 9.33 | 9.12 | 0.87 | 0.002229045 | 0.999976933 |
| 1500009C09Rik | XM_901684 | RIKEN cDNA 1500009C09 gene. transcript variant 2 (1500009C09Rik). | 12.62 | 12.42 | 0.87 | 0.002252338 | 0.999976933 |
| Prkcq | NM_008859 | protein kinase C. theta (Prkcq) | 8.62 | 8.36 | 0.84 | 0.002365774 | 0.999976933 |
| Dync1i1 | NM_010063 | dynein cytoplasmic 1 intermediate chain 1 (Dync1i1) | 10.53 | 10.28 | 0.85 | 0.002409405 | 0.999976933 |
| Cln3 | NM_001146311 | ceroid lipofuscinosis. neuronal 3. juvenile (Batten. Spielmeyer-Vogt disease) (Cln3). transcript variant 1 | 10.80 | 10.97 | 1.13 | 0.002449864 | 0.999976933 |
| Vat1l | NM_173016 | vesicle amine transport protein 1 homolog-like (T. californica) (Vat1l) | 14.00 | 13.79 | 0.86 | 0.00246407 | 0.999976933 |
| Crispld2 | NM_030209 | cysteine-rich secretory protein LCCL domain containing 2 (Crispld2) | 8.52 | 8.24 | 0.83 | 0.00248486 | 0.999976933 |
| D1Ertd622e | NM_133825 | DNA segment. Chr 1. ERATO Doi 622. expressed (D1Ertd622e) | 13.05 | 12.86 | 0.87 | 0.00250092 | 0.999976933 |
| Aqp4 | NM_009700 | aquaporin 4 (Aqp4) | 10.00 | 9.59 | 0.76 | 0.002622507 | 0.999976933 |
| Dync1i1 | NM_010063 | dynein cytoplasmic 1 intermediate chain 1 (Dync1i1) | 12.53 | 12.27 | 0.83 | 0.002686612 | 0.999976933 |
| Paqr7 | NM_027995 | progestin and adipoQ receptor family member VII (Paqr7) | 9.67 | 9.43 | 0.84 | 0.002742841 | 0.999976933 |
| BC030336 | NM_001164580 | cDNA sequence BC030336 (BC030336) | 12.48 | 12.30 | 0.89 | 0.002759113 | 0.999976933 |
| 1700071A11Rik | XM_973359 | RIKEN cDNA 1700071A11 gene (1700071A11Rik) | 9.36 | 9.10 | 0.84 | 0.00279515 | 0.999976933 |
| Pygl | NM_133198 | liver glycogen phosphorylase (Pygl) | 9.93 | 9.57 | 0.78 | 0.002809568 | 0.999976933 |
| Ces7 | NM_001003951 | carboxylesterase 7 (Ces7) | 5.65 | 5.44 | 0.86 | 0.002864528 | 0.999976933 |
| Cycs | NM_007808 | cytochrome c. somatic (Cycs). nuclear gene encoding mitochondrial protein | 13.61 | 13.46 | 0.90 | 0.002938562 | 0.999976933 |
| Tmem136 | NM_001034863 | transmembrane protein 136 (Tmem136). | 12.73 | 12.52 | 0.86 | 0.002967226 | 0.999976933 |
| Cops7a | NM_012003 | COP9 (constitutive photomorphogenic) homolog. subunit 7a (Arabidopsis thaliana) (Cops7a). transcript variant 1 | 13.70 | 13.53 | 0.89 | 0.003094076 | 0.999976933 |
| Chrna6 | NM_021369 | cholinergic receptor. nicotinic. alpha polypeptide 6 (Chrna6) | 13.15 | 12.87 | 0.83 | 0.003143173 | 0.999976933 |
| Alpl | NM_007431 | alkaline phosphatase. liver/bone/kidney (Alpl) | 12.27 | 12.01 | 0.83 | 0.003164741 | 0.999976933 |
| ENSMUST00000082319 | ENSMUST00000082319 | WD repeat domain 33 | 10.06 | 10.23 | 1.12 | 0.003200733 | 0.999976933 |
| Acot11 | NM_025590 | acyl-CoA thioesterase 11 (Acot11) | 10.03 | 9.78 | 0.84 | 0.00330161 | 0.999976933 |
| Fam71e2 | NM_172895 | family with sequence similarity 71. member E2 (Fam71e2) | 5.24 | 4.87 | 0.77 | 0.003307043 | 0.999976933 |
| Nacc2 | NM_001037098 | nucleus accumbens associated 2. BEN and BTB (POZ) domain containing (Nacc2). transcript variant 2 | 11.38 | 11.11 | 0.83 | 0.003434659 | 0.999976933 |
| Wbp4 | NM_018765 | WW domain binding protein 4 (Wbp4) | 9.28 | 9.18 | 0.93 | 0.003586293 | 0.999976933 |
| Fam196b | NM_001025382 | family with sequence similarity 196. member B (Fam196b) | 9.01 | 8.85 | 0.90 | 0.003606823 | 0.999976933 |
| Trim43b | XM_001480610 | predicted gene. EG666747 (EG666747). | 3.67 | 4.40 | 1.66 | 0.00371591 | 0.999976933 |
| Cav2 | NM_016900 | caveolin 2 (Cav2) | 9.11 | 8.74 | 0.77 | 0.003716208 | 0.999976933 |
| Spata5 | NM_001163511 | spermatogenesis associated 5 (Spata5). transcript variant 1 | 10.26 | 10.12 | 0.91 | 0.003748744 | 0.999976933 |
| A_55_P2140953 | A_55_P2140953 | Unknown | 14.80 | 14.66 | 0.91 | 0.003800666 | 0.999976933 |
| Anks1b | NM_001128086 | ankyrin repeat and sterile alpha motif domain containing 1B (Anks1b) | 8.08 | 7.85 | 0.85 | 0.00397653 | 0.999976933 |
| Ifi202b | NM_008327 | interferon activated gene 202B (Ifi202b). transcript variant 1. | 4.52 | 4.25 | 0.83 | 0.003994286 | 0.999976933 |
| Ablim2 | NM_177678 | actin-binding LIM protein 2 (Ablim2) | 10.24 | 10.05 | 0.88 | 0.004086898 | 0.999976933 |
| 4932416K20Rik | AK030040 | adult male testis cDNA. RIKEN full-length enriched library. clone:4932416K20 | 5.22 | 5.45 | 1.18 | 0.004110772 | 0.999976933 |
| Rpl23 | NM_022891 | ribosomal protein L23 (Rpl23) | 11.45 | 11.32 | 0.92 | 0.004190781 | 0.999976933 |
| Cav2 | NM_016900 | caveolin 2 (Cav2) | 9.15 | 8.76 | 0.76 | 0.004237035 | 0.999976933 |
| ENSMUST00000097361 | ENSMUST00000097361 | Putative uncharacterized protein | 6.02 | 6.25 | 1.17 | 0.004263388 | 0.999976933 |
| Nxt2 | NM_172782 | nuclear transport factor 2-like export factor 2 (Nxt2). transcript variant 1 | 11.90 | 11.68 | 0.86 | 0.004281393 | 0.999976933 |
| Ucn | NM_021290 | urocortin (Ucn) | 4.49 | 4.02 | 0.72 | 0.004314985 | 0.999976933 |
| Cav2 | NM_016900 | caveolin 2 (Cav2) | 9.17 | 8.77 | 0.76 | 0.004339765 | 0.999976933 |
| Gm9195 | XM_001479612 | similar to 3110045G13Rik protein (LOC668482) | 8.49 | 8.19 | 0.81 | 0.004343099 | 0.999976933 |
| Kcnh1 | NM_010600 | potassium voltage-gated channel. subfamily H (eag-related). member 1 (Kcnh1). transcript variant 1 | 6.07 | 5.76 | 0.81 | 0.004408405 | 0.999976933 |
| Dbp | NM_016974 | D site albumin promoter binding protein (Dbp) | 14.26 | 14.01 | 0.84 | 0.004466762 | 0.999976933 |
| Acpl2 | NM_153420 | acid phosphatase-like 2 (Acpl2) | 10.54 | 10.34 | 0.87 | 0.004480969 | 0.999976933 |
| Lrrc67 | NM_027033 | leucine rich repeat containing 67 (Lrrc67). transcript variant 1 | 8.54 | 8.30 | 0.85 | 0.004486922 | 0.999976933 |
| Tmem127 | NM_175145 | transmembrane protein 127 (Tmem127) | 14.04 | 13.95 | 0.94 | 0.004500787 | 0.999976933 |
| Sh3bgr | NM_015825 | SH3-binding domain glutamic acid-rich protein (Sh3bgr) | 9.11 | 8.82 | 0.82 | 0.004616881 | 0.999976933 |
| En1 | NM_010133 | engrailed 1 (En1) | 3.70 | 2.68 | 0.49 | 0.00479274 | 0.999976933 |
| Adarb1 | NM_001024837 | adenosine deaminase. RNA-specific. B1 (Adarb1). transcript variant 2 | 13.59 | 13.45 | 0.91 | 0.004968544 | 0.999976933 |
| B3gnt9 | NM_178879 | UDP-GlcNAc:betaGal beta-1.3-N-acetylglucosaminyltransferase 9 (B3gnt9) | 9.32 | 9.21 | 0.93 | 0.00498826 | 0.999976933 |
| ENSMUST00000077287 | ENSMUST00000077287 | Truncated cis-retinol/3alpha-hydroxysterol short-chain dehydrogenaseMCG17030 | 5.07 | 5.38 | 1.24 | 0.00500978 | 0.999976933 |
| Rybp | NM_019743 | RING1 and YY1 binding protein (Rybp) | 12.93 | 12.81 | 0.92 | 0.005051842 | 0.999976933 |
| 2410006H16Rik | NR_030738 | RIKEN cDNA 2410006H16 gene (2410006H16Rik). non-coding RNA | 12.20 | 12.00 | 0.87 | 0.005117745 | 0.999976933 |
| Ndrg4 | NM_145602 | N-myc downstream regulated gene 4 (Ndrg4) | 15.89 | 15.66 | 0.85 | 0.005133423 | 0.999976933 |
| Mmd2 | NM_175217 | monocyte to macrophage differentiation-associated 2 (Mmd2) | 12.40 | 12.28 | 0.92 | 0.005145472 | 0.999976933 |
| Cav2 | NM_016900 | caveolin 2 (Cav2) | 9.64 | 9.25 | 0.76 | 0.00519689 | 0.999976933 |
| Ngfr | NM_033217 | nerve growth factor receptor (TNFR superfamily. member 16) (Ngfr) | 11.47 | 11.25 | 0.85 | 0.005321404 | 0.999976933 |
| Zfp503 | NM_145459 | zinc finger protein 503 (Zfp503). | 9.43 | 9.09 | 0.79 | 0.005382397 | 0.999976933 |
| Pabpn1l | NM_001007462 | poly(A)binding protein nuclear-like 1 (Pabpnl1) | 5.84 | 5.55 | 0.82 | 0.005412312 | 0.999976933 |
| 4930579G22Rik | NM_026916 | RIKEN cDNA 4930579G22 gene (4930579G22Rik) | 9.45 | 9.31 | 0.91 | 0.005604349 | 0.999976933 |
| Fam171b | NM_175514 | family with sequence similarity 171. member B (Fam171b) | 13.99 | 13.73 | 0.84 | 0.00561929 | 0.999976933 |
| Tmem191c | NM_177473 | transmembrane protein 191C (Tmem191c) | 10.93 | 10.68 | 0.84 | 0.005694009 | 0.999976933 |
| Rhbdl3 | NM_139228 | rhomboid. veinlet-like 3 (Drosophila) (Rhbdl3) | 10.56 | 10.79 | 1.17 | 0.005761602 | 0.999976933 |
| Faim2 | NM_028224 | Fas apoptotic inhibitory molecule 2 (Faim2). transcript variant 1 | 10.98 | 10.72 | 0.83 | 0.005789341 | 0.999976933 |
| Cops7a | NM_012003 | COP9 (constitutive photomorphogenic) homolog. subunit 7a (Arabidopsis thaliana) (Cops7a). transcript variant 1 | 13.71 | 13.56 | 0.90 | 0.005931156 | 0.999976933 |
| Cacna1s | NM_001081023 | calcium channel. voltage-dependent. L type. alpha 1S subunit (Cacna1s). transcript variant 2 | 10.18 | 9.97 | 0.86 | 0.005994644 | 0.999976933 |
| Slc22a8 | NM_031194 | solute carrier family 22 (organic anion transporter). member 8 (Slc22a8). transcript variant 1 | 6.62 | 5.96 | 0.63 | 0.006032603 | 0.999976933 |
| Cops7a | NM_012003 | COP9 (constitutive photomorphogenic) homolog. subunit 7a (Arabidopsis thaliana) (Cops7a). transcript variant 1 | 13.42 | 13.28 | 0.90 | 0.006099232 | 0.999976933 |
